# Electroacupuncture for the prevention of perioperative neurocognitive disorder in elderly patients undergoing general anesthesia: a systematic review and meta-analysis

**DOI:** 10.3389/fmed.2026.1729153

**Published:** 2026-01-23

**Authors:** Changle Wu, Xuqiang Wei, Fei Luo, Jinyun Li, Yan Yin Joseph Kwan, Ke Wang, Jia Zhou

**Affiliations:** 1 Acupuncture Anesthesia Clinical Research Institute, Shanghai Acupuncture Clinical Research Center, Yueyang Hospital of Integrated Traditional Chinese and Western Medicine, Shanghai University of Traditional Chinese Medicine, Shanghai, China; 2 Department of Rehabilitation, Renmin Hospital, Hubei University of Medicine, Shiyan, Hubei, China; 3 School of Foreign Studies, Fuzhou University, Fuzhou, Fujian, China; 4 Faculty of Health and Life Sciences, University of Bristol, Bristol, United Kingdom

**Keywords:** elderly, electroacupuncture, meta-analysis, perioperative neurocognitive disorder, postoperative cognitive dysfunction, postoperative delirium

## Abstract

**Background:**

Perioperative neurocognitive disorder (PND) is a common complication following major surgery under general anesthesia, particularly among elderly patients, and adversely impacts postoperative recovery and quality of life. Although electroacupuncture (EA) has shown potential in preventing PND, conclusive evidence remains lacking. This study aimed to evaluate the effectiveness and safety of perioperative EA intervention for preventing PND in elderly patients undergoing general anesthesia surgery.

**Methods:**

We systematically searched eight electronic databases [PubMed, Embase, Web of Science, Cochrane Library, China National Knowledge Infrastructure (CNKI), Chongqing VIP Chinese Science and Technology Periodical Database (CQVIP), Wan Fang Database, and China Biology Medicine disc (CBM)] and three clinical trial registries from inception to March 16, 2025. Eligible studies were randomized controlled trials (RCTs) investigating perioperative EA for PND prevention in patients aged ≥ 60 years receiving general anesthesia. Control interventions included sham EA, standard care, or no intervention. Primary outcome was PND incidence. Secondary outcomes included neuropsychological assessment scores [Mini-Mental State Examination (MMSE) and Montreal Cognitive Assessment (MoCA)], inflammatory biomarkers [serum interleukin-1β (IL-1β), interleukin-6 (IL-6), and tumor necrosis factor-α (TNF-α) levels], neurological damage markers [serum neuron-specific enolase (NSE), S100 calcium-binding protein β (S100β) levels], and safety outcomes (incidence of adverse events). Two reviewers independently performed blind screening, data extraction, and risk-of-bias assessment using the Cochrane RoB 2 tool. Meta-analyses were conducted using RevMan 5.4, with random-effects or fixed-effect models applied based on heterogeneity (*I*^2^ statistics). The certainty of evidence was evaluated with the GRADE.

**Results:**

Twenty-six RCTs (*n* = 2,309) were included. Compared to the control groups, perioperative EA significantly reduced the incidence of PND (RR = 0.47, 95% CI: 0.42 to 0.54, *p* < 0.00001; *I*^2^ = 0%; moderate to low certainty), improved MMSE scores (MD = 1.92, 95% CI: 1.59 to 2.26, *p* < 0.00001; *I*^2^ = 96%; low to very low certainty), lowered serum IL-6 (SMD = −1.09, 95% CI: −1.73 to −0.44, *p* = 0.0010; *I*^2^ = 88%; very low certainty), IL-1β (SMD = −2.85, 95% CI: −5.32 to −0.39, *p* = 0.02; *I*^2^ = 99%; very low certainty), TNF-α (SMD = −2.64, 95% CI: −4.16 to −1.12, *p* = 0.0007; *I*^2^ = 98%; low certainty), and S100β (SMD = −1.56, 95% CI: −2.77 to −0.35, *p* = 0.01; *I*^2^ = 97%; low certainty) levels, and reduced adverse events (RR = 0.52, 95% CI: 0.37 to 0.72, *p* < 0.0001; *I*^2^ = 0%; moderate certainty). MoCA scores and serum NSE levels could not be meta-analyzed due to insufficient data.

**Conclusion:**

Perioperative EA intervention demonstrates significant clinical benefits in elderly patients undergoing general anesthesia surgery, effectively reducing PND incidence, improving cognitive function, attenuating neuroinflammation, and reducing neurological injury with a favorable safety profile. However, current evidence is constrained by methodological limitations, including potential selection bias and insufficient blinding in the included studies. Future rigorously designed multicenter RCTs with standardized EA featuring standardized EA protocols and long-term cognitive monitoring are needed to confirm its neuroprotective effects.

**Systematic review registration:**

https://www.crd.york.ac.uk/prospero/, identifier CRD420251035172.

## 1 Introduction

Perioperative neurocognitive disorder (PND), an updated diagnostic entity superseding the term postoperative cognitive dysfunction (POCD), encompasses newly acquired cognitive impairments in surgical patients. These manifest as deficits in memory, attention, executive function, language, and spatial orientation (1). According to current consensus nomenclature, PND integrates three clinical trajectories: preexisting cognitive impairment, postoperative delirium (POD), and POCD (1, 2). Consequently, in the postoperative context, PND includes both POCD and POD.

PND arises from the multifactorial interactions between predisposing vulnerabilities (e.g., advanced age) and precipitating insults (e.g., deep anesthesia, major surgical trauma) (1, 3, 4). The reported incidence of PND ranges from 12.0 to 25.8% (1) in general surgical populations, escalating to > 50% (1, 3) among patients aged ≥ 60 years. This condition significantly prolongs hospital stays, increases healthcare costs, elevates readmission risks, reduces quality of life, and is associated with higher long-term dementia incidence and mortality (5–8).

In the absence of disease-modifying pharmacotherapies for PND, clinical guidelines increasingly emphasize non-pharmacological prevention strategies (1, 3, 9). Electroacupuncture (EA) has emerged as a promising intervention, with perioperative application demonstrating potential to attenuate PND pathogenesis. Mechanistic studies indicate EA modulates neuroinflammation, synaptic plasticity, oxidative stress, microglial activation, neuronal apoptosis, and cerebral perfusion (10–14). Correspondingly, clinical trials report reduced PND incidence and enhanced cognitive recovery following EA in elderly patients undergoing knee arthroplasty, gastrectomy, and spinal surgery (15–19), with a favorable safety profile. Notwithstanding, existing systematic reviews (20–25) exhibit notable limitations: focusing narrowly on the effects of transcutaneous electrical acupoint stimulation (TEAS) and isolated outcomes (e.g., POCD or POD) rather than evaluating PND as a composite endpoint.

To address these gaps, we conducted a comprehensive systematic review and meta-analysis of randomized controlled trials (RCTs) investigating perioperative EA for PND prevention in elderly patients (≥ 60 years) undergoing general anesthesia. This study aimed to: (1) synthesize evidence on EA’s effectiveness in reducing PND incidence; (2) evaluate its effects on secondary outcomes, including inflammatory biomarkers and neurological recovery; and (3) critically appraise methodological rigor of existing studies, identifying limitations to inform future trial design. By integrating POCD and POD endpoints, this study provides a holistic evaluation of EA’s therapeutic potential and offers evidence-based recommendations for clinical practice and future research priorities.

## 2 Methods

### 2.1 Registration and reporting

This systematic review was conducted in accordance with the *Cochrane Handbook for Systematic Reviews of Interventions* (26) and is reported in compliance with the Preferred Reporting Items for Systematic reviews and Meta-Analyses (27). The study protocol was prospectively registered with PROSPERO (https://www.crd.york.ac.uk/prospero/, CRD420251035172) prior to data extraction.

### 2.2 Data sources and search strategy

A comprehensive search was conducted across eight electronic databases: PubMed, Embase, Cochrane Central Register of Controlled Trials (CENTRAL), Web of Science, China National Knowledge Infrastructure (CNKI), Wan Fang, Chongqing VIP Chinese Science and Technology Periodical Database (CQVIP), and China Biomedical Literature Database (CBM), from inception to March 16, 2025. To minimize selection bias, supplementary sources were explored, including https://ClinicalTrials.gov, the Chinese Clinical Trial Registry (ChiCTR), the International Traditional Medicine Clinical Trial Registry (ITMCTR), and gray literature (e.g., dissertations). Reference lists of included studies were manually screened to identify additional eligible trials.

The search strategy combined Medical Subject Headings (MeSH) and free-text terms across four domains: (1) Population: “aged,” “elderly,” “geriatric,” “perioperative neurocognitive disorder,” “postoperative cognitive dysfunction,” “delirium,” “cognitive decline.” (2) Intervention: “electroacupuncture,” “acupuncture,” “EA.” (3) Context: “general anesthesia,” “surgery,” “postoperative period.” (4) Study design: “randomized controlled trial,” “RCT.” Boolean operators (AND/OR/NOT) and truncation symbols (*) were applied to optimize sensitivity and specificity (see Supplementary Table S1 for full search syntax).

### 2.3 Eligibility criteria

#### 2.3.1 Inclusion criteria

(1) Design: Parallel-group RCTs in English or Chinese.
(2) Population: Patients aged ≥ 60 years undergoing elective surgery under general anesthesia, without preoperative cognitive impairment (MMSE/MoCA-confirmed).
(3) Intervention: Perioperative EA (preoperative, intraoperative, postoperative, or combined multi-phase), with no restrictions on acupoints selection, waveform, frequency, intensity, or duration. EA refers to the intervention where sterile acupuncture needles are first inserted subcutaneously into predefined acupoints (in line with Traditional Chinese Medicine acupoint localization standards) and retained at the target depth. Subsequently, the handles of the inserted needles are connected to an electroacupuncture device, which delivers continuous electrical stimulation to the acupoints via the needles.
(4) Comparators: sham EA (non-penetrating/non-acupoint stimulation), no intervention, or standard care.
(5) Outcomes: The primary outcome was the incidence of PND (defined as POCD or POD). POCD was diagnosed based on changes in MMSE scores before and after surgery, while POD was assessed using the Confusion Assessment Method (CAM) or the Nursing Delirium Screening Scale (Nu-DESC). Secondary outcomes included cognitive function (assessed by MMSE or MoCA scores), inflammatory biomarkers [serum interleukin-1*β* (IL-1β), interleukin-6 (IL-6), and tumor necrosis factor-α (TNF-α) levels], neurotoxicity markers [serum neuron-specific enolase (NSE) and S100 calcium-binding protein β (S100β) levels], and adverse events (AEs).

#### 2.3.2 Exclusion criteria

(1) non-RCTs (e.g., cohort studies, case reports, reviews).
(2) Patients with preexisting cognitive dysfunction according to the MMSE or MoCA, or those with cognitive impairment caused by neurodegenerative diseases such as Alzheimer’s disease, mild cognitive impairment, vascular dementia, mental illness, cranial brain trauma, stroke and other psychiatric conditions before surgery.
(3) Trials comparing EA parameters (e.g., waveform, frequency, intensity) without a non-EA control.
(4) The literatures with apparent errors in outcome measures or unclear diagnostic criteria for outcome events.
(5) The studies that did not assess cognitive function before surgery or studies with a sample size of less than 30 in the intervention or control group. The former criteria ensured baseline comparability and credible outcome interpretation, while the latter safeguarded statistical power.
(6) If the outcome data were duplicated, the older publications were excluded.

### 2.4 Study selection and data extraction

The EndNote X20 (Clarivate Analytics) was used for deduplication, and then two independent reviewers (CW and XW) screened the titles, abstracts and full texts of studies based on eligibility criteria, in order to eliminate irrelevant publications and retain eligible studies. Discrepancies were resolved by a third reviewer (KW). A standardized extraction template (28) was developed, capturing: (1) Study characteristics (first author, publication year, nationality, etc.); (2) Participant demographics (age, sex ratio, sample size, type of surgery, etc.); (3) Intervention details [acupoints, stimulation parameters (waveform, frequency, intensity), timing (pre-/intra-/postoperative), session duration]; (4) Outcomes (evaluation indicators, evaluation time, assessment tools, diagnostic criteria for outcome events). Missing data were requested from corresponding authors via email; unresolved omissions led to exclusion from quantitative synthesis.

### 2.5 Risk of bias assessment

Two investigators (CW and XW) independently assessed the risk of bias of included literatures according to the Version 2 of the Cochrane risk-of-bias tool for randomized trials (RoB 2) (29). The following contents were assessed: random sequence generation, allocation concealment, blinding of participants and personnel, blinding of outcome assessment, incomplete outcome data, selective reporting, and other bias. Each domain was rated as “low,” “high,” or “unclear” risk by two reviewers (CW and XW), with arbitration by KW.

### 2.6 Evidence quality assessment

Two researchers (CW and XW) independently assessed the evidence certainty for each critical outcome using the Grading of Recommendations, Assessments, Developments and Evaluations (GRADE) approach (30), yielding classifications of “high,” “moderate,” “low,” or “very low” confidence. Disagreements were resolved by KW.

### 2.7 Data synthesis and statistical analysis

RevMan version 5.4 (Cochrane Collaboration, Oxford, UK) was used for meta-analysis. Mean differences (MDs) or standardized mean differences (SMDs) with 95% confidence intervals (CI) for continuous variables, and relative risk (RR) with 95% CI for dichotomous variables. Heterogeneity was quantified via *I*^2^ statistic and Q test. *I*^2^ ≤ 50% and *p* ≥ 0.1 indicated acceptable heterogeneity, and a fixed-effect model was selected for meta-analysis. Conversely (*I*^2^ > 50% or *p* < 0.1), it indicated the presence of obvious heterogeneity, and a random-effects model was elected (31). Statistical significance was defined as two-tailed *p* < 0.05. The pre-specified subgroup analyses were conducted according to the EA intervention time, evaluation timepoints of outcome index, the number of EA intervention sessions, and surgical types to explore the reasons for heterogeneity. In addition, sensitivity analyses were conducted by excluding low-quality studies with high risk of bias per round to evaluate the outcome stability and investigate the sources of inconsistency among included literatures. When the number of included studies for an outcome indicator exceeded 10, funnel plot asymmetry test and Egger’s regression test were used to assess publication bias (32). *p* < 0.05 indicated a publication bias.

## 3 Results

### 3.1 Study identification

**Figure 1:**
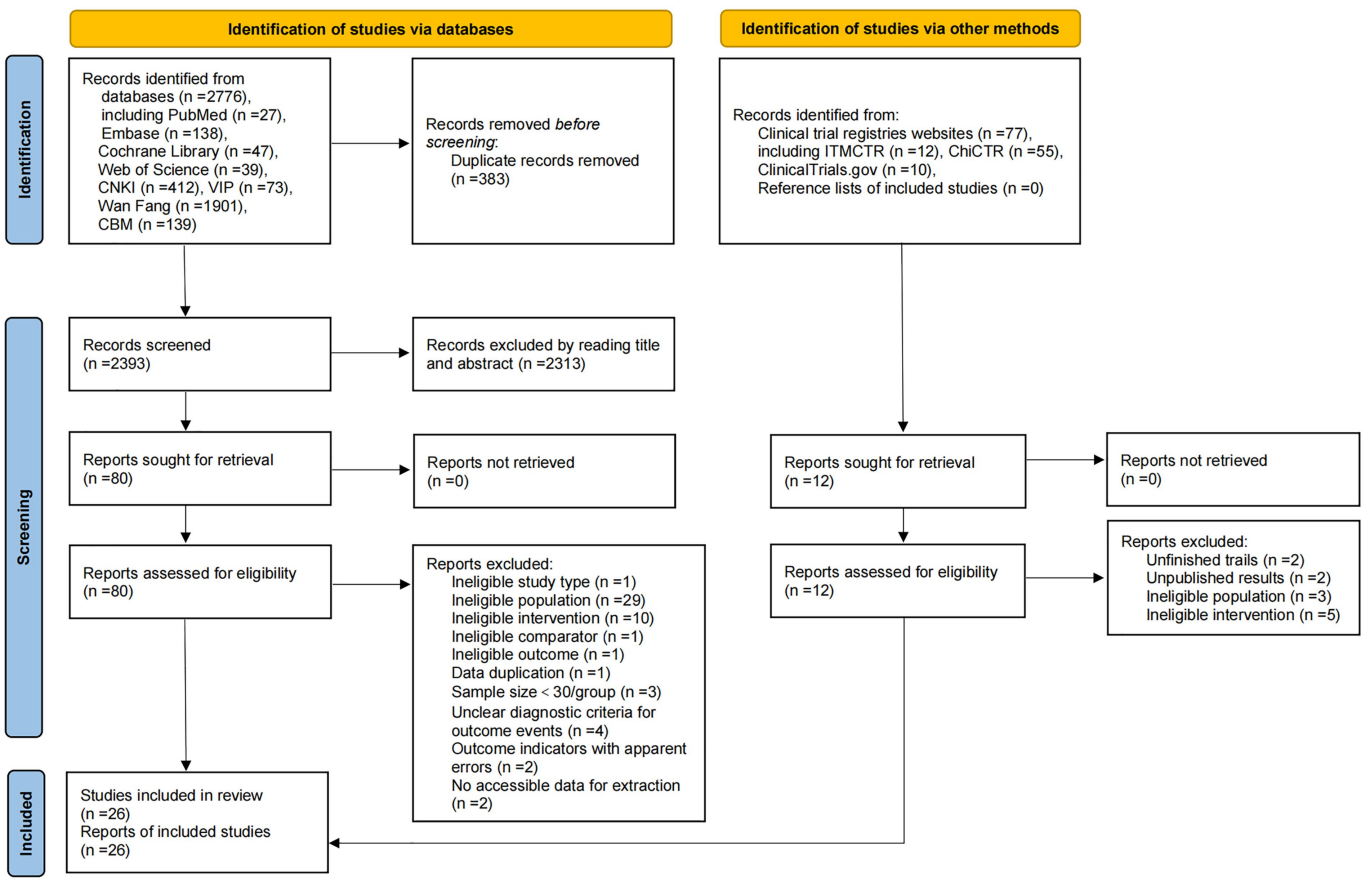
PRISMA flowchart for literature screening.

A total of 2,776 publications (2,525 Chinese and 251 English) were initially identified through database searches. After removing duplicates, the articles were screened by reading the titles, abstracts, and full texts, ultimately retaining 26 eligible studies (16, 18, 19, 33–55) for analysis. Supplementary searches of clinical trial registries (https://ClinicalTrials.gov, ChiCTR, ITMCTR) and reference lists of included studies retrieved no additional eligible publications. The selection process rigorously adhered to PRISMA guidelines, with a detailed flowchart outlining inclusion/exclusion decisions provided in Figure 1.

### 3.2 Study characteristics

All 26 included trials were two-arm parallel studies conducted in China and published between 2011 and 2023. A total of 2,309 participants were included, with 1,150 in the EA group and 1,159 in the control group. Subjects spanned multiple specialties: 11 orthopedic surgery (18, 19, 35, 37, 40, 42, 43, 45, 47, 52, 53, 55), six gastroenterological surgery (16, 33, 36, 38, 39, 49), two hepatobiliary surgery (50, 51), two urological surgery (44, 48), four mixed types (34, 37, 41, 42, 46), and one unspecified study (54). Regarding the EA intervention timing, seven studies used EA before surgery, 11 during surgery, three after surgery, one both before and during surgery, three both before and after surgery, and one combined EA before, during, and after surgery. The acupoints used with a frequency of 3 or more times were Baihui (GV20, 21 studies), Neiguan (PC6, 18 studies), Zusanli (ST36, 12 studies), Hegu (LI4, 8 studies), Shenting (GV24, 5 studies), Sanyinjiao and Taichong (SP6, LR3, 4 studies each), and Sishencong and Shenmen (EX-HN1, HT7, three studies each). The waveforms of EA intervention were mostly chosen as dense-disperse wave (21 articles), with the frequency often set at 2/100 Hz (nine articles), and the stimulation intensity was generally based on patient tolerance (17 articles). The specific characteristics of the included literatures are shown in Supplementary Table S2.

### 3.3 Risk of bias

**Figure 2:**
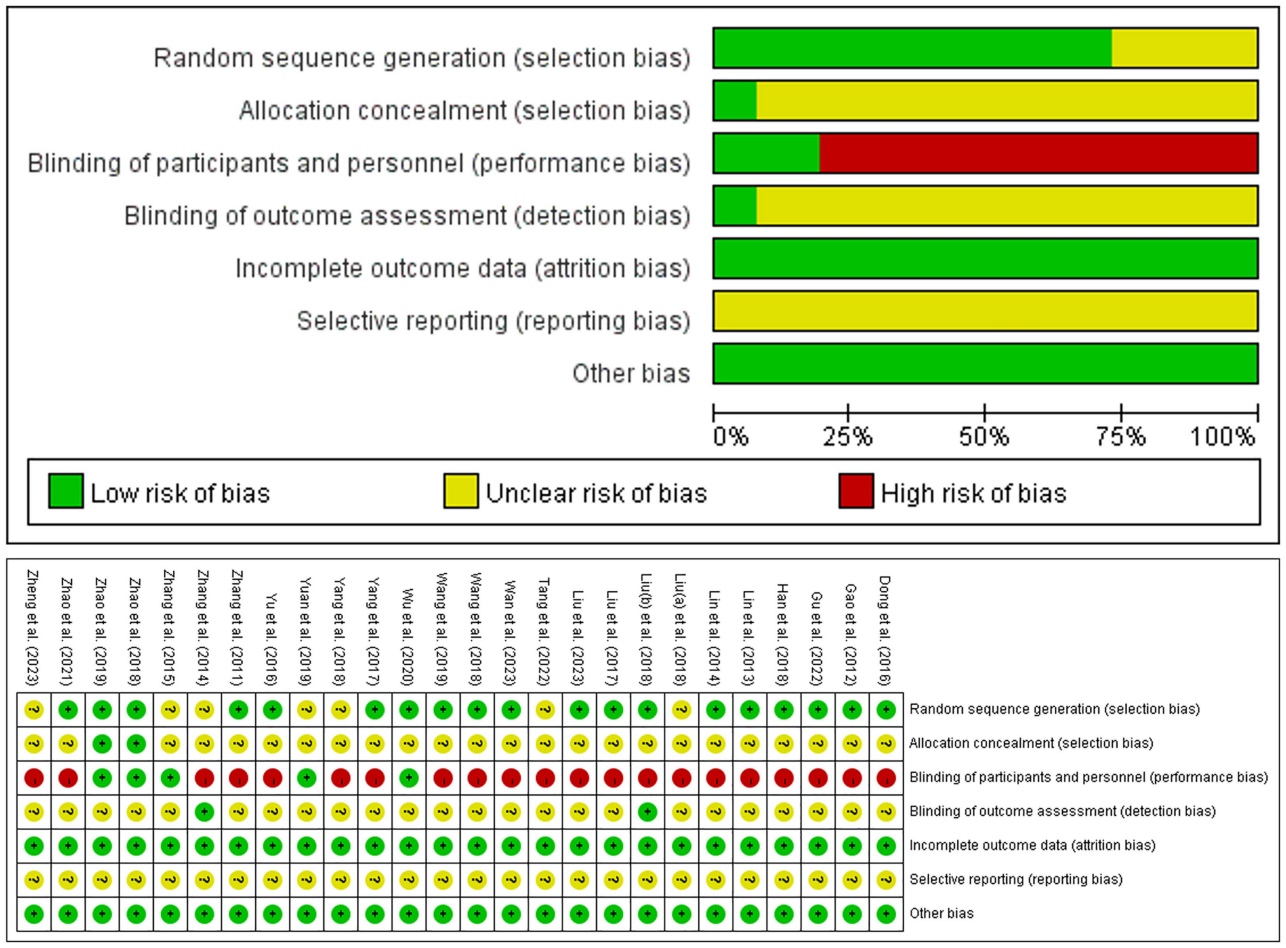
The risk of bias graph.

Among the 26 included studies, 18 studies (16, 18, 19, 33–42, 44–47, 52, 54) (69.2%) used a random number table, one study (49) (3.9%) employed the coin toss, and seven studies (37, 42, 43, 48, 50, 51, 53, 55) (26.9%) stated “randomized” without method specification. Only two studies (18, 19) (7.7%) adequately described the methods of allocation concealment. Twenty-one studies (16, 33–45, 47–49, 52–55) (80.8%) were rated as high risk of bias due to lack of blinding of participants and personnel, whereas the remaining five studies (18, 19, 46, 50, 51) (19.2%) demonstrated low risk through implementation of sham EA controls. Only two studies (37, 42, 53) reported outcome blinding assessment (7.7%), others were unclear. All included studies (100%) demonstrated low risk of incomplete outcome data. Unavailable protocols (100%) precluded assessment of the selective reporting bias. No other sources of bias were identified across the studies (100%) (Figure 2).

### 3.4 Primary outcome

**Figure 3:**
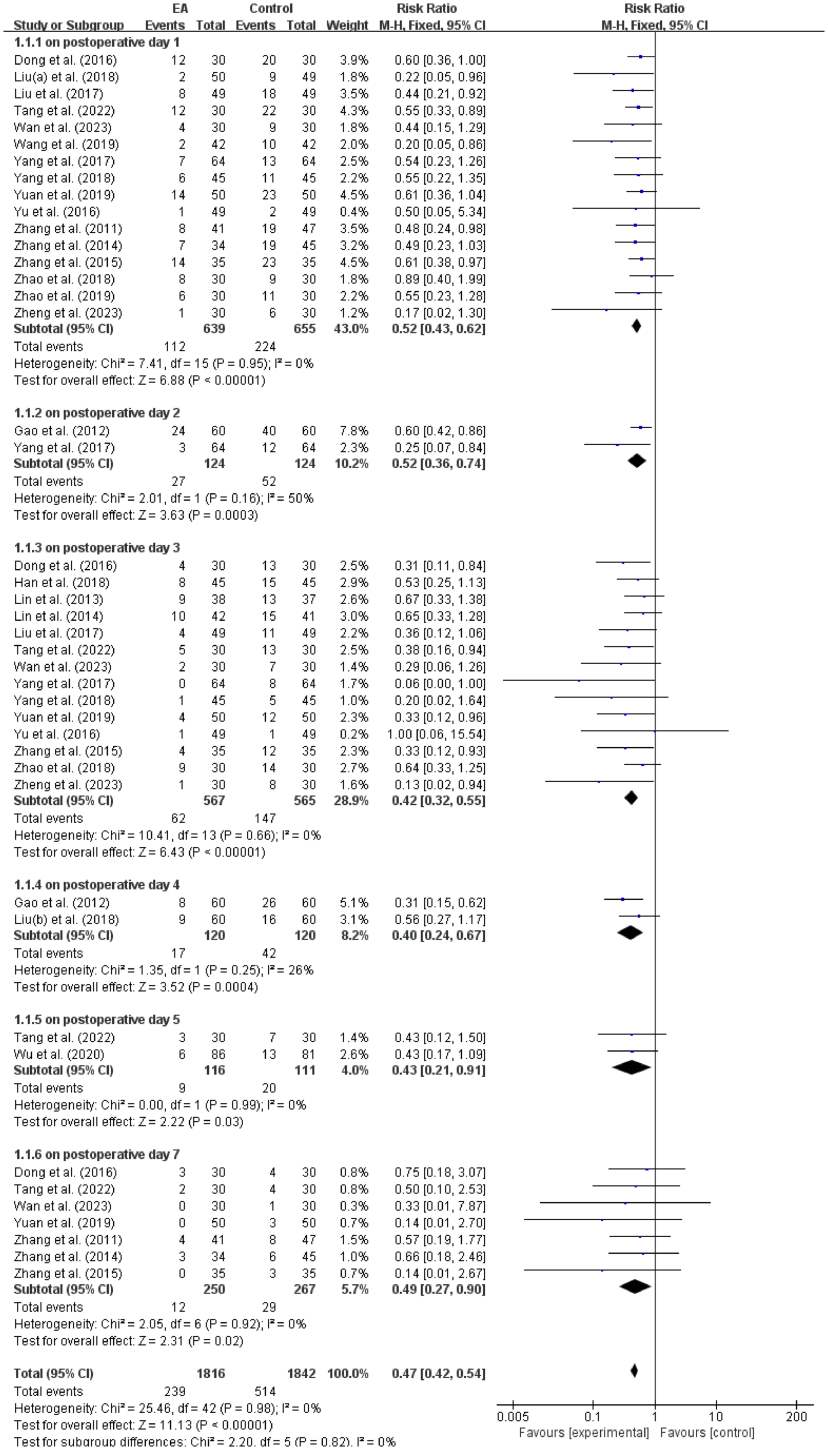
Meta-analysis and forest plot for the incidence of PND at different periods. PND, perioperative neurocognitive disorder.

Twenty-two studies (18, 19, 33, 34, 36–39, 41–53, 55); (*n* = 3,658) reported PND incidence after excluding one study (54) with unclear diagnostic criteria. Timepoints with single-study reporting (at 6, and 12 h postoperatively/on postoperative days 6 and 14) were excluded from pooled analysis to maintain statistical reliability. Perioperative EA significantly reduced overall PND incidence versus controls (RR = 0.47, 95% CI: 0.42 to 0.54, *p* < 0.00001; *I*^2^ = 0%). Subgroup analysis by postoperative day (POD) revealed consistent reductions. POD 1 (16 studies): RR = 0.52, 95% CI: 0.43 to 0.62, *p* < 0.00001; *I*^2^ = 0%. POD 2 (two studies): RR = 0.52, 95% CI: 0.36 to 0.74, *p* = 0.0003; *I*^2^ = 50%. POD 3 (14 studies): RR = 0.42, 95% CI: 0.32 to 0.55, *p* < 0.00001; *I*^2^ = 0%. POD 4 (two studies): RR = 0.40, 95% CI: 0.24 to 0.67, *p* = 0.0004; *I*^2^ = 26%. POD 5 (two studies): RR = 0.43, 95% CI: 0.21 to 0.91, *p* = 0.03; *I*^2^ = 0%. POD 7 (seven studies): RR = 0.49, 95% CI: 0.27 to 0.90, *p* = 0.02; *I*^2^ = 0% (all analyses used fixed-effect model; Figure 3).

### 3.5 Secondary outcomes

#### 3.5.1 MMSE scores

**Figure 4:**
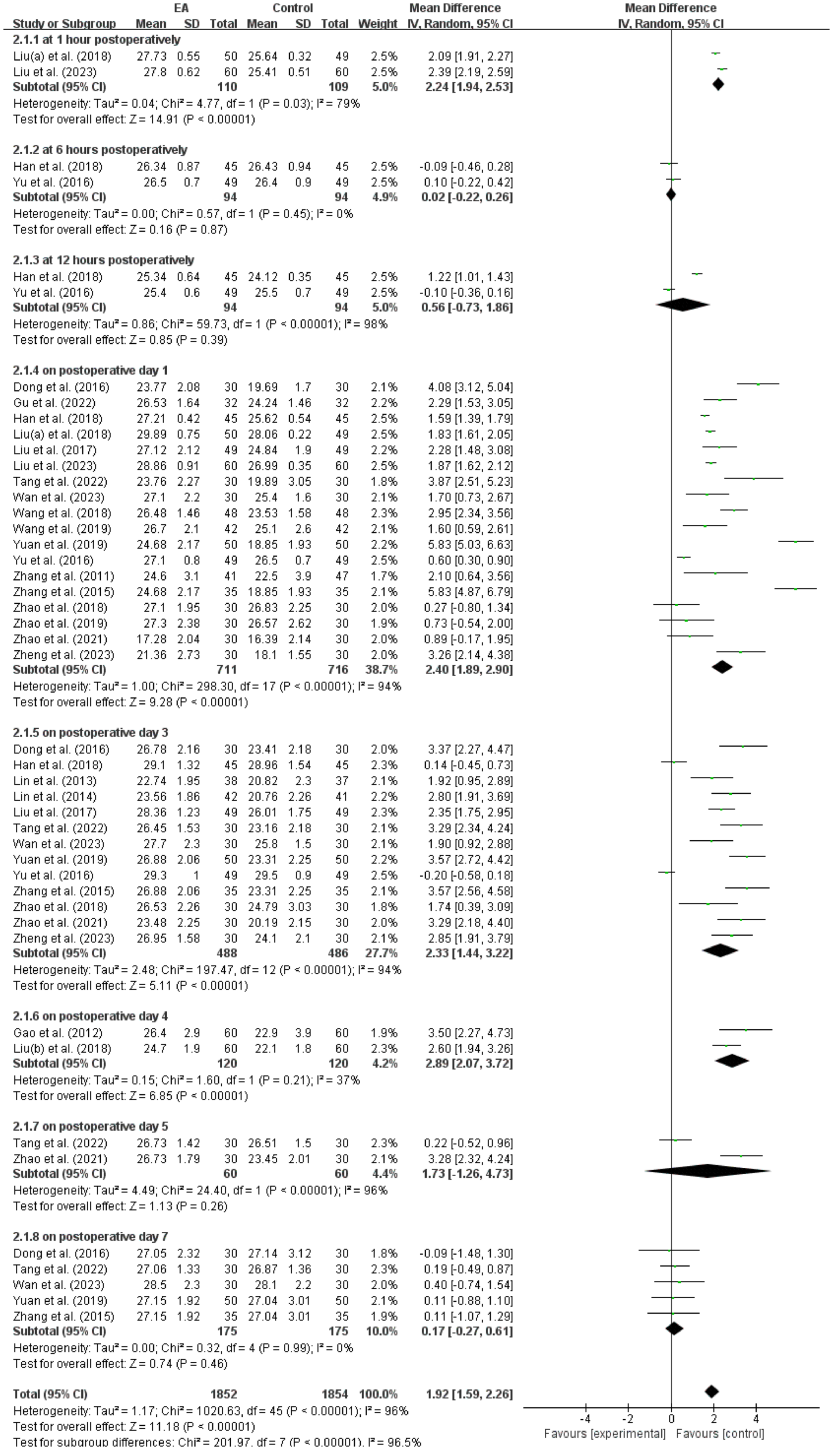
Meta-analysis and forest plot for the MMSE scores at different periods. MMSE, Mini-Mental State Examination.

Twenty-two studies (16, 18, 19, 33–44, 49–52, 54, 55) assessed MMSE scores. Meta-analysis (random-effects model; *I*^2^ = 96%, *p* < 0.00001) showed significant EA-associated improvements at 1 h postoperatively (MD = 2.24, 95% CI: 1.94 to 2.53, *p* < 0.00001; *I*^2^ = 79%), POD 1 (MD = 2.40, 95% CI: 1.89 to 2.90, *p* < 0.00001; *I*^2^ = 94%), POD 3 (MD = 2.33, 95% CI: 1.44 to 3.22, *p* < 0.00001; *I*^2^ = 94%), and POD 4 (MD = 2.89, 95% CI: 2.07 to 3.72, *p* < 0.00001; *I*^2^ = 37%). No significant differences at 6 h, 12 h, POD 5, or POD 7 (Figure 4).

#### 3.5.2 Serum IL-6 levels

A total of five studies (41, 43, 45, 53, 54) quantified serum IL-6 levels using enzyme-linked immunosorbent assay (ELISA). The results showed that EA exhibited a significant reduction in serum IL-6 levels on POD 1 (SMD = −1.09, 95% CI: −1.73 to −0.44, *p* = 0.0010; *I*^2^ = 88%, random-effects model). No significant intergroup difference at the end of surgery (Supplementary Table S3).

#### 3.5.3 Serum IL-1β levels

Four studies (19, 37, 41, 42, 53) reported serum IL-1β levels utilizing ELISA after excluding one (18) with unspecified methodology. EA significantly reduced IL-1β levels on POD 1 (SMD = −2.85, 95% CI: −5.32 to −0.39, *p* = 0.02; *I*^2^ = 99%), but no significant difference was observed at the end of surgery (Supplementary Table S3).

#### 3.5.4 Serum TNF-α levels

Seven studies reported serum TNF-α levels, among which six (19, 37, 41–43, 45, 53) used ELISA, while one study (18) was excluded from meta-analysis due to unclear detection method. The results revealed that EA significantly decreased serum TNF-α levels on POD 1 (SMD = −2.64, 95% CI: −4.16 to −1.12, *p* = 0.0007; *I*^2^ = 98%). However, no significant intergroup difference on POD 3 (Supplementary Table S3).

#### 3.5.5 Serum NSE levels

Of three studies (37, 40, 42, 48) reporting serum NSE levels, one (40) was excluded for methodological inconsistency (chemiluminescence vs. ELISA) and two (37, 42, 48) due to substantial heterogeneity [surgical types (urological vs. orthopedic/oncologic), EA intervention timing (preoperative vs. intraoperative), and EA stimulation frequencies (2/100 Hz vs. 4/20 Hz)], precluding quantitative synthesis.

#### 3.5.6 Serum S100β levels

Eleven studies (19, 36–38, 40, 42, 43, 46–48, 52, 53) reported serum S100β levels. Four studies were excluded from meta-analysis: two (36, 47) with unspecified detection methods and timepoints, one (52) with unavailable results, and one (40) using inconsistent method (fluorescence immunochromatography vs. ELISA). Compared with the control group, EA exhibited a remarkable reduction in S100β levels on POD 1 (SMD = −1.56, 95% CI: −2.77 to −0.35, *p* = 0.01; *I*^2^ = 97%), while no significant difference at the end of operation (Supplementary Table S3).

### 3.6 Subgroup analyses

Given the limited number of available studies, we conducted subgroup analyses exclusively for the PND incidence on POD 1 and POD 3, MMSE scores on POD 1 and POD 3, and serum levels of IL-6, IL-1β, TNF-α, and S100β on POD 1. These analyses were stratified according to predefined characteristics: EA intervention timing, comparators, surgical categories, and EA sessions. The results are presented in Supplementary Table S4.

The EA-related reduction in PND incidence on POD 1 and POD 3 was independent of intervention timing. Preoperative (POD 1: RR = 0.55, 95% CI: 0.39 to 0.77, *p* = 0.0004, *I*^2^ = 0%; POD 3: RR = 0.46, 95% CI: 0.26 to 0.83, *p* = 0.01, *I*^2^ = 0%), intraoperative (POD 1: RR = 0.42, 95% CI: 0.28 to 0.63, *p* < 0.0001, *I*^2^ = 0%; POD 3: RR = 0.52, 95% CI: 0.37 to 0.75, *p* = 0.0003, *I*^2^ = 0%), postoperative (POD 1: RR = 0.61, 95% CI: 0.43 to 0.87, *p* = 0.006, *I*^2^ = 0%; POD 3: RR = 0.33, 95% CI: 0.16 to 0.70, *p* = 0.004, *I*^2^ = 0%), preoperative-postoperative combined EA (POD 1: RR = 0.49, 95% CI: 0.32 to 0.75, *p* = 0.001, *I*^2^ = 0%; POD 3: RR = 0.22, 95% CI: 0.10 to 0.48, *p* = 0.0002, *I*^2^ = 24%) consistently demonstrated significant lower PND incidence. Moreover, the reduction in PND incidence on POD 1 and POD 3 was consistent regardless of comparators, demonstrating significant effectiveness against both sham EA (POD 1: RR = 0.64, 95% CI: 0.47 to 0.86, *p* = 0.004, *I*^2^ = 0%; POD 3: RR = 0.45, 95% CI: 0.27 to 0.74, *p* = 0.002, *I*^2^ = 0%) and no-intervention controls (POD 1: RR = 0.46, 95%CI: 0.36 to 0.59, *p* < 0.00001, *I*^2^ = 0%; POD 3: RR = 0.44, 95% CI: 0.32 to 0.60, *p* < 0.00001, *I*^2^ = 0%). The operation-stratified analysis revealed that EA significantly reduced PND incidence on POD 1 for orthopedic surgery (RR = 0.50, 95% CI: 0.38 to 0.66, *p* < 0.00001; *I*^2^ = 0%) and gastrointestinal surgery (RR = 0.57, 95% CI: 0.43 to 0.75, *p* < 0.0001; *I*^2^ = 0%), whereas no significant superiority in urological procedure (RR = 0.50, 95% CI: 0.25 to 1.00, *p* = 0.05; *I*^2^ = 0%), potentially attributable to limited study availability (*n* = 2). For PND incidence on POD 3, EA exhibited consistent reductions in orthopedic (RR = 0.36, 95% CI: 0.21 to 0.59, *p* < 0.0001; *I*^2^ = 47%), urological (RR = 0.25, 95% CI: 0.07 to 0.84, *p* = 0.03; *I*^2^ = 0%), and gastrointestinal surgery (RR = 0.47, 95% CI: 0.34 to 0.65, *p* < 0.00001; *I*^2^ = 0%), suggesting neuroprotective benefit independent of surgical types.

EA intervention time is a key source of heterogeneity of MMSE scores on POD 1 and POD 3 (subgroup differences: *p* = 0.008; *p* = 0.002). The combination of preoperative and postoperative EA showed a significant improvement (MD = 3.51, 95% CI: 2.64 to 4.37, *p* < 0.00001; *I*^2^ = 0%) in MMSE scores on POD 1. Preoperative (MD = 1.59, 95%CI: 0.48 to 2.69, *p* = 0.005), intraoperative (MD = 1.85, 95% CI: 1.38 to 2.33, *p* < 0.00001), or postoperative (MD = 4.20, 95% CI: 1.17 to 7.22, *p* = 0.007) EA revealed moderate improvement in MMSE scores on POD 1, but the high heterogeneity (*I*^2^ = 83%; *I*^2^ = 93%; *I*^2^ = 97%) suggested potential confounding factors, such as surgical types, anesthesia drugs, patient demographics or EA parameters. Further homogeneous studies are needed to validate these findings and clarify the optimal timing of EA for cognitive protection. Preoperative (MD = 1.84, 95% CI: 1.05 to 2.64, *p* < 0.00001), postoperative (MD = 3.50, 95% CI: 2.94 to 4.06, *p* < 0.00001), preoperative-postoperative combined EA (MD = 3.07, 95% CI: 2.40 to 3.74, *p* < 0.00001) exhibited a significant improvement and consistent effects (*I*^2^ = 0%) in MMSE scores on POD 3. Notably, postoperative EA intervention demonstrated superior effectiveness (MD = 3.50). Intraoperative EA showed mild improvement and more variable effects in MMSE scores on POD 3 (MD = 1.69, 95% CI: 0.45 to 2.92, *p* = 0.007; *I*^2^ = 95%), warranting further investigation.

The number of EA sessions may be a significant and partial source of heterogeneity in serum IL-6 and IL-1β levels on POD 1 (between-subgroup difference: *p* = 0.05; *p* < 0.00001). Multiple EA sessions (≥ 2) produced a marked reduction in IL-6 (SMD = −1.65, 95% CI: −2.07 to −1.23, *p* < 0.00001; *I*^2^ = 0%) and IL-1β (SMD = −5.37, 95%CI: −6.09 to −4.65, *p* < 0.00001; *I*^2^ = 17%) levels on POD 1, while single EA session demonstrated no intergroup difference. These findings indicate that repeated EA interventions may exert cumulative anti-inflammatory effects.

EA intervention timing significantly contributed to heterogeneity in serum TNF-α levels on POD 1 (*p* < 0.00001). Intraoperative EA demonstrated a significant reduction in serum TNF-α levels on POD1 (SMD = −1.07, 95% CI: −1.43 to −0.72, *p* < 0.00001; *I*^2^ = 22%). Surgical type substantially influenced outcome heterogeneity of serum S100β levels on POD 1 (*p* < 0.0001). While EA intervention did not significantly decrease S100β levels on POD 1 in orthopedic surgery patients (SMD = −0.21, 95%CI: −0.56 to 0.13, *p* = 0.22; *I*^2^ = 33%), it demonstrated beneficial effects across multiple surgical types (SMD = −3.71, 95% CI: −5.91 to −1.51, *p* = 0.0009; *I*^2^ = 96%). This variability likely reflects differences in the degree of cerebral injury associated with distinct surgical procedures.

### 3.7 Sensitivity analysis

To assess the robustness of the findings, sensitivity analyses were performed by sequentially excluding low-quality studies with high risk of implementation bias. For the incidence of PND on postoperative days 1 and 3, statistical significance and effect direction remained unchanged after exclusion of high-bias studies, indicating high result stability. After excluding the study by Gu et al. (35), the statistical significance shifted in MMSE scores on POD 1 (from *p* < 0.00001 to *p* = 0.05), suggesting result instability due to low-quality studies. Exclusion of Han et al.’ study (36) altered the significance level in MMSE scores on POD 3 (from *p* < 0.00001 to *p* = 0.07), further indicating methodological bias influenced the outcome (Supplementary Table S5).

### 3.8 Incidence of adverse events

Eight studies (16, 18, 19, 33–35, 40, 52) reported adverse events, including nausea and vomiting, minor acupuncture-induced hematoma, headache, dizziness, and venous thrombosis. No significant heterogeneity was observed across studies (*I*^2^ = 0%, *p* = 0.42), and a fixed-effect model was applied for analysis. The pooled results demonstrated that the EA group had a significantly lower incidence of adverse events compared to the control group (RR = 0.52, 95% CI: 0.37 to 0.72, *p* < 0.0001; *I*^2^ = 0%) (Supplementary Figure 1).

### 3.9 Publication bias

**Figure 5:**
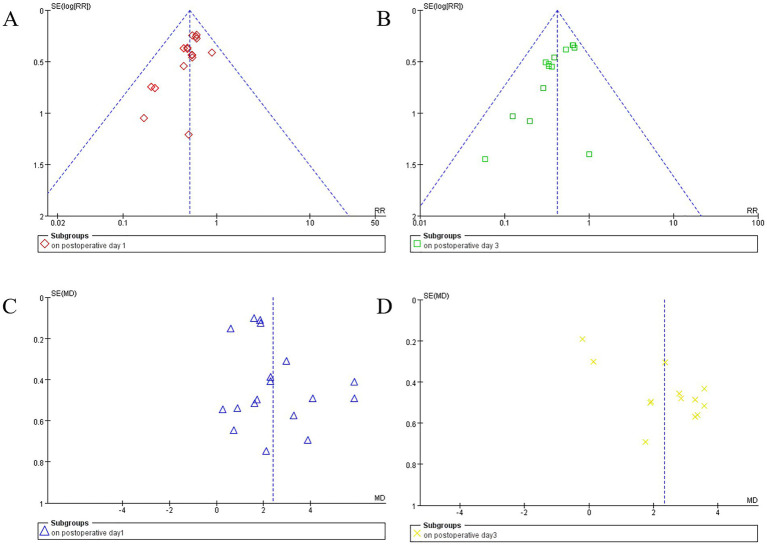
Funnel plots display study data by subgroups. Plot **(A)** (red diamonds) and **(B)** (green squares) show the funnel plot for perioperative neurocognitive disorder (PND) incidence on postoperative day 1and day 3. Plots **(C)** (blue triangles) and **(D)** (yellow crosses) illustrate mean differences in MMSE scores for postoperative day 1 and day 3, respectively. Vertical dashed lines indicate summary effect estimates.

For postoperative day 1 PND incidence (16 studies), postoperative day 3 PND incidence (14 studies), and postoperative day 1 MMSE scores (18 studies), the funnel plots appeared symmetrical, and Egger’s test yielded no significant publication bias (*p* = 0.052, *p* = 0.063, and *p* = 0.087, respectively). However, for postoperative day 3 MMSE scores (13 studies), the funnel plot exhibited noticeable asymmetry, and Egger’s test indicated significant publication bias (*p* = 0.001) (Figure 5).

### 3.10 Evidence quality

The overall certainty of evidence regarding the effectiveness of EA on relevant outcomes is summarized in Table 1. Moderate certainty evidence indicates that EA is associated with reduced incidence of PND on POD 1 and POD 3, and lower incidence of adverse events. Low or very low certainty evidence suggests that EA may be linked to decreased PND incidence on POD 2, POD 4, POD 5, and POD 7; improved postoperative cognitive function; and alleviated peripheral inflammatory responses (IL-6, IL-1β, and TNF-α) and cerebral injury severity (S100β).

**Table 1:** The overall evidence quality for outcome measure.

Outcomes	No. of studies	No. of participants		Quality assessment					Effect size (95% CI)	Certainty
		EG	CG	Risk of bias	Inconsistency	Indirectness	Imprecision	Other consideration		
Incidence of PND										
On postoperative day 1	16	639	655	Downgraded^a^	Not downgraded	Not downgraded	Not downgraded	Not downgraded	RR 0.52 (0.43 ~ 0.62)	Moderate
On postoperative day 2	2	124	124	Downgraded^a^	Not downgraded	Not downgraded	Downgraded^c^	Not downgraded	RR 0.52 (0.36 ~ 0.74)	Low
On postoperative day 3	14	567	565	Downgraded^a^	Not downgraded	Not downgraded	Not downgraded	Not downgraded	RR 0.42 (0.32 ~ 0.55)	Moderate
On postoperative day 4	2	120	120	Downgraded^a^	Not downgraded	Not downgraded	Downgraded^c^	Not downgraded	RR 0.40 (0.24 ~ 0.67)	Low
On postoperative day 5	2	116	111	Downgraded^a^	Not downgraded	Not downgraded	Downgraded^c^	Not downgraded	RR 0.43 (0.21 ~ 0.91)	Low
On postoperative day 7	7	250	267	Downgraded^a^	Not downgraded	Not downgraded	Downgraded^c^	Not downgraded	RR 0.49 (0.27 ~ 0.90)	Low
MMSE scores										
At 1 h postoperatively	2	110	109	Downgraded^a^	Downgraded^b^	Not downgraded	Downgraded^c^	Not downgraded	MD 2.24 (1.94 ~ 2.53)	Very low
At 6 h postoperatively	2	94	94	Downgraded^a^	Not downgraded	Not downgraded	Downgraded^c^	Not downgraded	MD 0.02 (−0.22 ~ 0.26)	Low
At 12 h postoperatively	2	94	94	Downgraded^a^	Downgraded^b^	Not downgraded	Downgraded^c^	Not downgraded	MD 0.56 (−0.73 ~ 1.86)	Very low
On postoperative day 1	18	711	716	Downgraded^a^	Downgraded^b^	Not downgraded	Not downgraded	Not downgraded	MD 2.40 (1.89 ~ 2.90)	Low
On postoperative day 3	13	488	486	Downgraded^a^	Downgraded^b^	Not downgraded	Not downgraded	Downgraded^d^	MD 2.33 (1.44 ~ 3.22)	Very low
On postoperative day 4	2	120	120	Downgraded^a^	Not downgraded	Not downgraded	Downgraded^c^	Not downgraded	MD 2.89 (2.07 ~ 3.72)	Low
On postoperative day 5	2	60	60	Downgraded^a^	Downgraded^b^	Not downgraded	Downgraded^c^	Not downgraded	MD 1.73 (−1.26 ~ 4.73)	Very low
On postoperative day 7	5	175	175	Downgraded^a^	Not downgraded	Not downgraded	Downgraded^c^	Not downgraded	MD 0.17 (−0.27 ~ 0.61)	Low
Serum IL-6 levels										
At the end of surgery	2	83	94	Downgraded^a^	Downgraded^b^	Not downgraded	Downgraded^c^	Not downgraded	SMD -0.72 (−1.89 ~ 0.44)	Very low
On postoperative day 1	5	185	196	Downgraded^a^	Downgraded^b^	Not downgraded	Downgraded^c^	Not downgraded	SMD -1.09 (−1.73 ~ −0.44)	Very low
Serum IL-1β levels										
At the end of surgery	2	83	94	Downgraded^a^	Downgraded^b^	Not downgraded	Downgraded^c^	Not downgraded	SMD -1.04 (−2.69 ~ 0.61)	Very low
On postoperative day 1	4	173	184	Downgraded^a^	Downgraded^b^	Not downgraded	Downgraded^c^	Not downgraded	SMD -2.85 (−5.32 ~ −0.39)	Very low
Serum TNF-α levels										
On postoperative day 1	6	245	256	Downgraded^a^	Downgraded^b^	Not downgraded	Not downgraded	Not downgraded	SMD -2.64 (−4.16 ~ −1.12)	Low
On postoperative day 3	2	60	60	Downgraded^a^	Downgraded^b^	Not downgraded	Downgraded^c^	Not downgraded	SMD -3.70 (−7.96 ~ 0.55)	Very low
Serum S100β levels										
At the end of surgery	4	208	212	Downgraded^a^	Downgraded^b^	Not downgraded	Downgraded^c^	Not downgraded	SMD -0.58 (−1.29 ~ 0.13)	Very low
On postoperative day 1	6	275	280	Downgraded^a^	Downgraded^b^	Not downgraded	Not downgraded	Not downgraded	SMD -1.56 (−2.77 ~ −0.35)	Low
Incidence of adverse events	8	331	337	Downgraded^a^	Not downgraded	Not downgraded	Not downgraded	Not downgraded	RR 0.52 (0.37, 0.72)	Moderate

EG, electroacupuncture group; CG, control group; CI, confidence intervals; PND, perioperative neurocognitive disorder; RR, relative risk; MMSE: Mini-Mental State Examination; MD, mean difference; IL-6, interleukin-6; SMD, standardized mean difference; IL-1β, interleukin-1β; TNF-α, tumor necrosis factor-α; S100β, S100 calcium-binding protein β.

a Downgraded by 1 level due to high or unclear risk of bias.

b Downgraded by 1 level due to substantial heterogeneity (*I*^2^ > 50%).

c Downgraded by 1 level due to the inconformity of optimal information size (OIS) criterion and/or 95% CI included a null value (RR = 1 or MD/SMD = 0).

d Downgraded by 1 level due to publication bias (*p* < 0.05).

## 4 Discussion

### 4.1 Key findings

Perioperative neurocognitive disorder (PND) imposes substantial clinical and socioeconomic burdens on elderly surgical patients, adversely affecting both immediate recovery and long-term outcomes (1, 56). This meta-analysis demonstrates that perioperative electroacupuncture (EA) significantly reduces PND incidence (POD 1, 2, 3, 4, 5, and 7) and enhances early cognitive recovery (MMSE at 1 h postoperatively, POD1, POD3, and POD 4) with a favorable safety profile. Low-to-moderate certainty evidence indicates EA’s neuroprotective potential through attenuation of systemic inflammation (reduced IL-1β, IL-6, TNF-α) and mitigation of neurological injury (lowered S100β) on POD 1. Crucially, PND reduction on POD 1 and POD 3 remained robust across EA timing, comparator types, and surgical categories, underscoring broad clinical applicability.

Subgroup analyses elucidated the critical influence of EA intervention timing and session frequency on efficacy. Combined preoperative-postoperative EA significantly improved MMSE scores on POD 1 (*p* < 0.00001), whereas postoperative-only EA enhanced MMSE scores on POD 3 (*p* < 0.00001). These findings support prioritizing either combined or postoperative-only EA as optimal timing strategies for cognitive protection in elderly patients under general anesthesia. A dose–response relationship was observed between EA sessions and anti-inflammatory effects, validating multi-session protocols (≥ 2 sessions) for maximal suppression of postoperative inflammation (serum IL-1β/IL-6 reduction; *p* < 0.00001).

Given inherent variability in anesthetic protocols and surgical trauma, procedure-specific subgroup analyses revealed differential neuroprotective effects of EA. Serum S100β reduction varied significantly by surgical category, suggesting procedure-related brain injury severity modulates EA efficacy. Sensitivity analysis sequentially excluding nonblinded trials demonstrated compromised robustness of MMSE improvements on PODs 1 and 3, indicating potential overestimation of EA effects in unblinded studies due to measurement bias or placebo effects. These results emphasize the necessity of rigorous blinding and standardized outcome assessments.

### 4.2 Advancement beyond prior research

This work represents the first comprehensive evaluation of EA for PND prevention specifically in elderly patients under general anesthesia, overcoming limitations of prior reviews focused on transcutaneous electrical acupoint stimulation (TEAS) (20–25), mixed anesthesia techniques (15), or heterogeneous populations (57). Through predefined subgroup analyses and sensitivity testing, we identified previously overlooked heterogeneity sources and established a multidimensional evidence chain integrating clinical outcomes (PND incidence), cognitive metrics (MMSE), and mechanistic biomarkers (inflammatory/neurological markers), thereby elucidating EA’s “intervention-efficacy-mechanism” relationship.

### 4.3 Mechanistic insights and superiorities

**Figure 6:**
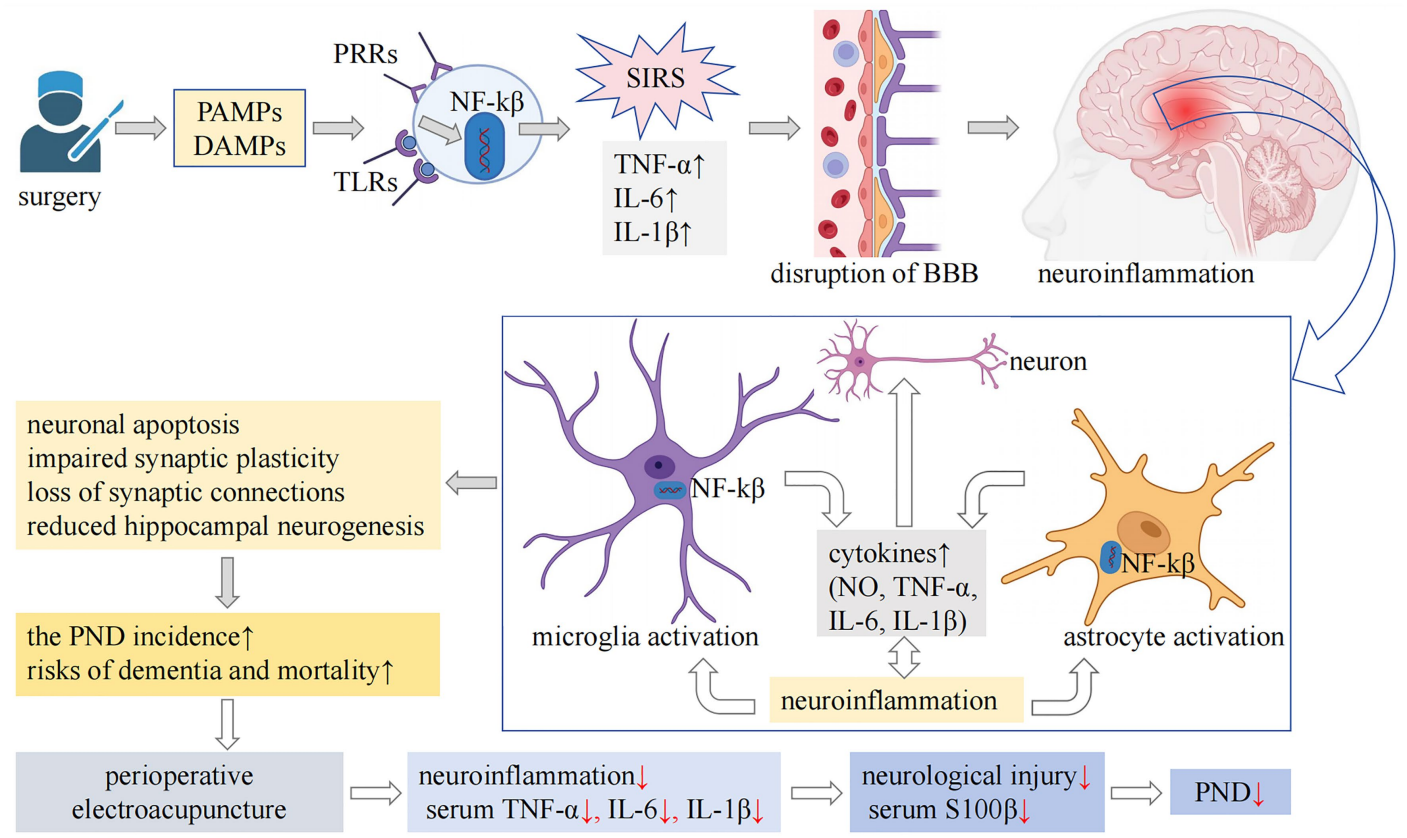
The mechanisms underlying PND and the potential pathways through which EA may exert preventive effects are complex. Surgical trauma and associated stress can result in cellular damage, prompting the release of intracellular immunogenic compounds, including PAMPs and DAMPs, into the extracellular milieu. These molecules interact with PRRs, such as TLRs, subsequently activating the NF-κ*β* signaling pathway. This activation leads to the secretion of proinflammatory cytokines, including TNF-α, IL-1β, and IL-6, thereby initiating a SIRS. SIRS is associated with increased permeability of the blood–brain barrier, a key feature of neuroinflammation. Additionally, lymphocytes recruited from peripheral sites infiltrate the central nervous system, activating microglia and astrocytes, which further intensifies neuroinflammatory processes. Persistent neuroinflammation is detrimental, as it contributes to neuronal dysfunction and apoptosis, impairs hippocampal neurogenesis, disrupts synaptic plasticity, and leads to synaptic loss. These pathological alterations may contribute to postoperative cognitive dysfunction and are linked to an increased long-term risk of dementia and mortality. Our findings indicate that perioperative EA intervention may have neuroprotective and cognitive-preserving effects. This is likely achieved through the mitigation of the systemic inflammatory response, as evidenced by reduced serum levels of IL-1β, IL-6, and TNF-α, and the attenuation of neural injury, as reflected by decreased serum S100β levels. Consequently, this intervention may lower the risk of PND. PND, perioperative neurocognitive disorder; EA, electroacupuncture; PAMPs, pathogen-associated molecular patterns; DAMPs, damage-associated molecular patterns; PRRs, pattern recognition receptors; TLRs, Toll-like receptors; NF-κβ, nuclear factor-κβ; TNF-α, tumor necrosis factor-α; IL-1β, interleukin-1β; IL-6, interleukin-6; SIRS, systemic inflammatory response syndrome; BBB, blood–brain barrier; NO, nitric oxide; S100β, S100 calcium-binding protein β.

Current evidence indicates that surgical trauma and stress-induced systemic inflammatory response syndrome can promote and exacerbate neuroinflammation through various mechanisms, including increased permeability of the blood–brain barrier and activation of glial cells. This neuroinflammatory cascade subsequently results in synaptic dysfunction, neuronal impairment and death, disrupted hippocampal neurogenesis, and compromised synaptic plasticity. Ultimately, these alterations contribute to the development of PND (58), as illustrated in Figure 6. EA’s neuroprotection aligns with established biological pathways. Preclinical evidence indicates acupuncture modulates neuroinflammation and systemic inflammatory responses (10, 59), enhances synaptic plasticity (10), inhibits microglial activation (13, 14), prevents neuronal apoptosis (14), and improves cognitive function (10, 13, 14, 60, 61). Neuroimaging studies further demonstrated acupuncture-mediated activation of cognition-related brain regions (e.g., prefrontal cortex) and strengthened functional neural connectivity (10, 60, 61). Our results suggest that perioperative EA may enhance cognitive function by potentially by mitigating the systemic inflammatory response and reducing neural damage, thereby decreasing the incidence of PND (Figure 6).

Beyond direct neuroprotection, EA mitigates preoperative anxiety, reduces intraoperative anesthetic requirements, and attenuates surgical stress responses—collectively contributing to PND prevention (1, 62, 63). Furthermore, EA harnesses the synergistic advantages of acupoint specificity, electrical stimulation, and cognitive protection. Unlike pharmacological interventions, which often exhibit limited efficacy and are associated with inherent side effects, EA offers a favorable safety profile, minimal invasiveness, and cost-effectiveness, significantly enhancing its appropriateness for perioperative application.

### 4.4 Limitations and future directions

Despite promising results, several limitations merit consideration: Firstly, all included studies were conducted in China, limiting generalizability to diverse populations with varying cultural acceptance of EA. Secondly, high statistical heterogeneity (*I*^2^ = 79–98%) arose from variability in EA parameters (waveforms, frequencies), surgical procedures, and outcome assessment protocols. Thirdly, inadequate blinding (80.8% of studies) and allocation concealment (92.3%) undermine internal validity. Lastly, EA-related adverse events (e.g., hematoma, syncope) were inconsistently documented.

To address these gaps, we advocate for: (1) Multicenter RCTs: Stratified by surgical type and patient demographics, adhering to Consolidated Standards of Reporting Trials (CONSORT) (64) and Standards for Reporting Interventions in Clinical Trials of Acupuncture (STRICTA) (65) guidelines. (2) Standardized Protocols: Explicit reporting of EA parameters (e.g., waveform: 2/100 Hz; intensity: patient tolerance-adjusted) and anesthesia regimens. (3) Extended Follow-up: Longitudinal assessments (≥30 days) to evaluate sustained cognitive benefits. (4) Mechanistic Studies: Integration of neuroimaging and biomarker analyses to clarify multimodal neuroprotective pathways. (5) Rigorous Controls: Implementation of sham acupuncture per Sham Acupuncture Reporting Guidelines and Checklist for Clinical Trials (SHARE) (66) to isolate EA-specific effects. Future trials should either focus on single surgical types to minimize heterogeneity or conduct stratified analyses to delineate surgery-specific EA efficacy. Protocol optimization accounting for these factors is critical for enhancing EA’s therapeutic potential.

## 5 Conclusion

Perioperative electroacupuncture demonstrates robust clinical efficacy in reducing PND incidence, enhancing early cognitive recovery, and attenuating biomarkers of neuroinflammation (IL-1β, IL-6, TNF-α) and neurological injury (S100β) among elderly surgical patients. Despite methodological limitations inherent in extant literature, consistent benefits across prespecified subgroups support EA’s utility as a perioperative adjunct. Future high-quality, multicenter trials employing standardized EA protocols, diverse populations, and mechanistic depth are imperative to consolidate EA’s role in evidence-based perioperative care.

## Data Availability

The original contributions presented in the study are included in the article/Supplementary material, further inquiries can be directed to the corresponding authors.
