# Supplementary material for: Electroacupuncture for the prevention of perioperative neurocognitive disorder in elderly patients undergoing general anesthesia: a systematic review and meta-analysis
Source: Front Med (Lausanne). 2026 Jan 23;13:1729153. doi: 10.3389/fmed.2026.1729153 (PMC12876244; doi:10.3389/fmed.2026.1729153)
Supplement: Supplementary file 1 [file Supplementary_file_1.docx]

***Supplementary Material***

Contents

**Supplementary Table S1.** The detailed search strategy. **1**

**Supplementary Table S2.** Characteristics of included RCTs. **8**

**Supplementary Table S3.** Meta-analysis for the serum IL-6, IL-1β, TNF-α, and S100β levels at different periods. **14**

**Supplementary Table S4.** Subgroup analyses. **15**

**Supplementary Table S5.** Sensitivity analysis for PND incidence and MMSE scores. **17**

**Supplementary Figure 1.** Meta-analysis and forest plot for the incidence of adverse events. **18**

**Table S1.** The detailed search strategy.

| **Electronic databases** | **Search** | **Search strategy** | **Results** |
| --- | --- | --- | --- |
| **PubMed** | #1 | (cognition) OR (neurocognitive disorders) OR (cognitive dysfunction) OR (cognition disorders) OR (delirium) [MeSH Terms] | 27 |
|  | #2 | (cognitive disorders) OR (cognitive impairment) OR (cognitive decline) OR (cognition dysfunction) OR (cognition impairment) OR (cognition decline) OR (cognit*) [Title/Abstract] |  |
|  | #3 | #1 OR #2 |  |
|  | #4 | (postoperative period) OR (anesthesia recovery period) OR (postop*) OR (postoperative*) OR (postoperative) OR (postsurgical*) OR (post anesthesia) OR (after surgery) OR (post-surgery) [Title/Abstract] |  |
|  | #5 | #3 AND #4 |  |
|  | #6 | (postoperative cognitive complications) [MeSH Terms] |  |
|  | #7 | (delayed neurocognitive recovery) OR (perioperative neurocognitive disorder) OR (perioperative cognitive disorder) OR (POCD) OR (POD) OR (PND) [Title/Abstract] |  |
|  | #8 | #5 OR #6 OR #7 |  |
|  | #9 | (acupuncture) OR (electroacupuncture) OR (acupuncture therapy) [MeSH Terms] |  |
|  | #10 | (aged) [MeSH Terms] |  |
|  | #11 | (elderly) OR (older) OR (geriatric) [Title/Abstract] |  |
|  | #12 | #10 OR #11 |  |
|  | #13 | (randomized controlled trial) OR (controlled clinical trial) OR (random*) OR (randomization) OR (randomly) [Title/Abstract] |  |
|  | #14 | #8 AND #9 AND #12 AND #13 |  |
| **Cochrane** | #1 | ((neurocognitive disorders) OR (cognitive dysfunction) OR (cognition disorders) OR (cognitive disorders) OR (cognitive impairment) OR (cognitive decline) OR (cognition dysfunction) OR (cognition impairment) OR (cognition decline) OR (cognit*) OR (delirium)):ti,ab,kw | 47 |
|  | #2 | MeSH descriptor: [Cognition] explode all trees |  |
| **Electronic databases**  **Supplementary Table S1.** (Continued) | **Search** | **Search strategy** | **Results** |
| **Cochrane** | #3 | MeSH descriptor: [Neurocognitive Disorders] explode all trees | 47 |
|  | #4 | MeSH descriptor: [Cognitive Dysfunction] explode all trees |  |
|  | #5 | MeSH descriptor: [Cognition Disorders] explode all trees |  |
|  | #6 | MeSH descriptor: [Delirium] explode all trees |  |
|  | #7 | #1 OR #2 OR #3 OR #4 OR #5 OR #6 |  |
|  | #8 | ((postoperative period) OR (anesthesia recovery period) OR (postop*) OR (postoperative*) OR (postoperative) OR (postsurgical*) OR (post anesthesia) OR (after surgery) OR (post-surgery)):ti,ab,kw |  |
|  | #9 | MeSH descriptor: [Postoperative Period] explode all trees |  |
|  | #10 | MeSH descriptor: [Anesthesia Recovery Period] explode all trees |  |
|  | #11 | #8 OR #9 OR #10 |  |
|  | #12 | #7 AND #11 |  |
|  | #13 | ((postoperative cognitive complications) OR (delayed neurocognitive recovery) OR (perioperative neurocognitive disorder) OR (perioperative cognitive disorder) OR (POCD)):ti,ab,kw |  |
|  | #14 | MeSH descriptor: [Postoperative Cognitive Complications] explode all trees |  |
|  | #15 | #12 OR #13 OR #14 |  |
|  | #16 | ((acupuncture) OR (electroacupuncture) OR (EA)):ti,ab,kw |  |
|  | #17 | MeSH descriptor: [Acupuncture] explode all trees |  |
|  | #18 | MeSH descriptor: [Acupuncture Therapy] explode all trees |  |
|  | #19 | MeSH descriptor: [Electroacupuncture] explode all trees |  |
|  | #20 | #16 OR #17 OR #18 OR #19 |  |
|  | #21 | MeSH descriptor: [aged] explode all trees |  |
|  | #22 | ((elderly) OR (older) OR (geriatric)):ti,ab,kw |  |
|  | #23 | #21 OR #22 |  |
|  | #24 | ((randomized controlled trial) OR (controlled clinical trial) OR (random*) OR (randomization) OR (randomly)):ti,ab,kw |  |
| **Electronic databases**  **Supplementary Table S1.** (Continued) | **Search** | **Search strategy** | **Results** |
| **Cochrane** | #25 | MeSH descriptor: [Randomized Controlled Trial] explode all trees | 47 |
|  | #26 | MeSH descriptor: [Controlled Clinical Trial] explode all trees |  |
|  | #27 | #24 OR #25 OR #26 |  |
|  | #28 | #15 AND #20 AND #23 AND #27 |  |
| **Web of science** | #1 | TS=(“neurocognitive disorders” OR “cognitive dysfunction” OR “cognition disorders” OR “cognitive disorders” OR “cognitive impairment” OR “cognitive decline” OR “cognition dysfunction” OR “cognition impairment” OR “cognition decline” OR “cognit*” OR “delirium”) | 39 |
|  | #2 | TS=(“postoperative period” OR “anesthesia recovery period” OR “postop*” OR “postoperative*” OR “postoperative” OR “postsurgical*” OR “post anesthesia” OR “after surgery” OR “post-surgery”) |  |
|  | #3 | #1 AND #2 |  |
|  | #4 | TS=(“postoperative cognitive complications” OR “delayed neurocognitive recovery” OR “perioperative neurocognitive disorder” OR “perioperative cognitive disorder” OR “POCD” OR “POD” OR “PND”) |  |
|  | #5 | #3 OR #4 |  |
|  | #6 | TS=(“acupuncture” OR “electroacupuncture” OR “EA”) |  |
|  | #7 | TS=(“aged” OR “elderly” OR “older” OR “geriatric”) |  |
|  | #8 | TS=(“randomized controlled trial” OR “controlled clinical trial” OR “random*” OR “randomization” OR “randomly”) |  |
|  | #9 | #5 AND #6 AND #7 AND #8 |  |
| **Embase** | #1 | 'cognition'/exp OR cognition | 138 |
|  | #2 | 'disorders of higher cerebral function'/exp OR 'disorders of higher cerebral function' OR (('disorders'/exp OR disorders) AND of AND higher AND cerebral AND ('function'/exp OR function)) |  |
|  | #3 | 'cognitive defect'/exp OR 'cognitive defect' OR (cognitive AND defect) |  |
|  | #4 | 'delirium'/exp OR delirium |  |
|  | #5 | 'neurocognitive disorders'/exp OR 'neurocognitive disorders' OR (neurocognitive AND ('disorders'/exp OR disorders)) |  |
|  | #6 | 'cognitive dysfunction'/exp OR 'cognitive dysfunction' OR (cognitive AND dysfunction) |  |
|  | #7 | 'cognition disorders'/exp OR 'cognition disorders' OR (('cognition'/exp OR cognition) AND ('disorders'/exp OR |  |
| **Electronic databases**  **Supplementary Table S1.** (Continued) | **Search** | **Search strategy** | **Results** |
| **Embase** | #7 | disorders)) | 138 |
|  | #8 | 'cognitive disorders'/exp OR 'cognitive disorders' OR (cognitive AND ('disorders'/exp OR disorders)) |  |
|  | #9 | 'cognitive impairment'/exp OR 'cognitive impairment' OR (cognitive AND ('impairment'/exp OR impairment)) |  |
|  | #10 | 'cognitive decline'/exp OR 'cognitive decline' OR (cognitive AND ('decline'/exp OR decline)) |  |
|  | #11 | 'cognition dysfunction' OR (('cognition'/exp OR cognition) AND dysfunction) |  |
|  | #12 | 'cognition impairment' OR (('cognition'/exp OR cognition) AND ('impairment'/exp OR impairment)) |  |
|  | #13 | 'cognition decline' OR (('cognition'/exp OR cognition) AND ('decline'/exp OR decline)) |  |
|  | #14 | cognit* |  |
|  | #15 | #1 OR #2 OR #3 OR #4 OR #5 OR #6 OR #7 OR #8 OR #9 OR #10 OR #11 OR #12 OR #13 OR #14 |  |
|  | #16 | 'postoperative period'/exp OR 'postoperative period' OR (postoperative AND period) |  |
|  | #17 | 'anesthetic recovery'/exp OR 'anesthetic recovery' OR (('anesthetic'/exp OR anesthetic) AND ('recovery'/exp OR recovery)) |  |
|  | #18 | 'anesthesia recovery period'/exp OR 'anesthesia recovery period' OR (('anesthesia'/exp OR anesthesia) AND ('recovery'/exp OR recovery) AND period) |  |
|  | #19 | #16 OR #17 OR #18 |  |
|  | #20 | #15 AND #19 |  |
|  | #21 | 'postoperative cognitive dysfunction'/exp OR 'postoperative cognitive dysfunction' OR (postoperative AND cognitive AND dysfunction) |  |
|  | #22 | 'postoperative cognitive complications'/exp OR 'postoperative cognitive complications' OR (postoperative AND cognitive AND ('complications'/exp OR complications)) |  |
|  | #23 | 'delayed neurocognitive recovery'/exp OR 'delayed neurocognitive recovery' OR (delayed AND neurocognitive AND ('recovery'/exp OR recovery)) |  |
|  | #24 | 'perioperative neurocognitive disorder'/exp OR 'perioperative neurocognitive disorder' OR (perioperative AND neurocognitive AND ('disorder'/exp OR disorder)) |  |
| **Electronic databases**  **Supplementary Table S1.** (Continued) | **Search** | **Search strategy** | **Results** |
| **Embase** | #25 | 'perioperative cognitive disorder' OR (perioperative AND cognitive AND ('disorder'/exp OR disorder)) | 138 |
|  | #26 | pocd |  |
|  | #27 | #21 OR #22 OR #23 OR #24 OR #25 OR #26 |  |
|  | #28 | #20 OR #27 |  |
|  | #29 | 'acupuncture'/exp OR acupuncture |  |
|  | #30 | 'electroacupuncture'/exp OR electroacupuncture |  |
|  | #31 | ea |  |
|  | #32 | #29 OR #30 OR #31 |  |
|  | #33 | 'randomized controlled trial'/exp OR 'randomized controlled trial' OR (randomized AND controlled AND ('trial'/exp OR trial)) |  |
|  | #34 | 'controlled clinical trial'/exp OR 'controlled clinical trial' OR (controlled AND ('clinical'/exp OR clinical) AND ('trial'/exp OR trial)) |  |
|  | #35 | random* |  |
|  | #36 | 'randomization'/exp OR randomization |  |
|  | #37 | randomly |  |
|  | #38 | #33 OR #34 OR #35 OR #36 OR #37 |  |
|  | #39 | 'aged'/exp OR 'aged' |  |
|  | #40 | 'elderly'/exp OR elderly |  |
|  | #41 | 'older adult'/exp OR 'older adult' OR (older AND ('adult'/exp OR adult)) |  |
|  | #42 | 'geriatric'/exp OR geriatric |  |
|  | #43 | #39 OR #40 OR #41 OR #42 |  |
|  | #44 | #28 AND #32 AND #38 AND #43 |  |
| **CBM** | #1 | 针刺 OR 针刺疗法OR 电针 OR 电针疗法 OR “EA”（使用快速检索） | 139 |
|  | #2 | 认知 OR 认知障碍 OR 认知功能障碍 OR 神经认知障碍 OR 认知功能损害 OR 认知能力下降 OR 认 |  |
| **Electronic databases**  **Supplementary Table S1.** (Continued) | **Search** | **Search strategy** | **Results** |
| **CBM** | #2 | 知下降 OR 术后认知并发症 OR 认知恢复延迟 OR 谵妄 OR 精神错乱 OR 躁动 OR “POCD” OR “POD” OR “PND”（使用快速检索） | 139 |
|  | #3 | 围手术期 OR 麻醉恢复期 OR 术后（使用快速检索） |  |
|  | #4 | 随机对照试验 OR 临床对照试验 OR 临床试验 OR 多中心研究 OR 随机对照 OR 随机 OR 疗效观察（使用快速检索） |  |
|  | #5 | 老年 OR 老人 OR 高龄 OR 60岁（使用快速检索） |  |
|  | #6 | #1 AND #2 AND #3 AND #4 AND #5 |  |
| **CNKI** | #1 | (SU=(“针刺”+“针刺疗法”+“电针”+“电针疗法”+“EA”) OR TKA=(“针刺”+“针刺疗法”+“电针”+“电针疗法”+“EA”)) AND (SU=(“认知”+“认知障碍”+“认知功能障碍”+“神经认知障碍”+“认知损害”+“认知下降”+“认知减退”+“术后认知并发症”+“记忆障碍”+“认知恢复延迟”+“谵妄”+“精神错乱”+“躁动”+“PND”+“POD”+“POCD”) OR TKA=(“认知”+“认知障碍”+“认知功能障碍”+“神经认知障碍”+“认知损害”+“认知下降”+“认知减退”+“术后认知并发症”+“记忆障碍”+“认知恢复延迟”+“谵妄”+“精神错乱”+“躁动”+“PND”+“POD”+“POCD”)) AND (TKA=(“术后”+“围手术期”+“麻醉恢复期”)) AND (TKA=(“随机对照试验”+“临床对照试验”+“临床试验”+“随机对照”+“随机”+“多中心”+“疗效观察”))AND (TKA=(“老年”+“老人”+“高龄”+“60岁”)) | 412 |
| **Wan Fang** | #1 | (题名或关键词:(“针刺” OR “针刺疗法” OR “电针” OR “电针疗法” OR “EA”) OR 摘要:(“针刺” OR “针刺疗法” OR “电针” OR “电针疗法” OR “EA”)) AND (题名或关键词:(“认知障碍” OR “认知功能障碍” OR “神经认知障碍” OR “认知损害” OR “认知下降” OR “认知减退” OR “术后认知并发症” OR “认知恢复延迟” OR “谵妄” OR “POCD” OR “POD” OR “PND”) OR 摘要:(“认知障碍” OR “认知功能障碍” OR “神经认知障碍” OR “认知损害” OR “认知下降” OR “认知减退” OR “术后认知并发症” OR “认知恢复延迟” OR “谵妄” OR “POCD” OR “POD” OR “PND”)) AND (题名或关键词:(“术后” OR “围手术期” OR “麻醉恢复期”) AND (全部:(“临床试验” OR “随机对照” OR “随机” OR “临床研究” OR “疗效观察”)) AND (题名或关键词:(“老年” OR “老人” OR “高龄” OR “60岁”)) | 1901 |
| **Electronic databases** | **Search** | **Search strategy** | **Results** |
| **VIP** | #1 | (M=(针刺 OR 针刺疗法 OR 电针 OR 电针疗法 OR “EA”) OR R=(针刺 OR 针刺疗法 OR 电针 OR 电针疗法 OR “EA”)) AND (M=(认知 OR 认知障碍 OR 认知功能障碍 OR 神经认知障碍 OR 认知损害 OR 认知下降 OR 认知减退 OR 术后认知并发症 OR 认知恢复延迟 OR 谵妄 OR “POCD” OR “POD” OR “PND”) OR R=(认知 OR 认知障碍 OR 认知功能障碍 OR 神经认知障碍 OR 认知损害 OR 认知下降 OR 认知减退 OR 术后认知并发症 OR 认知恢复延迟 OR 谵妄 OR “POCD” OR “POD” OR “PND”)) AND (M=(术后 OR 围手术期 OR 麻醉恢复期)) AND (U=(随机对照试验 OR 临床对照试验 OR 临床试验 OR 随机对照 OR 随机 OR 多中心 OR 疗效观察)) AND (M=(老年 OR 老人 OR 高龄 OR “60岁”)) | 73 |
| [**http://www.chictr.org.cn/**](http://www.chictr.org.cn/) | #1 | 注册题目= (认知 OR 认知损害 OR 认知下降 OR 认知减退 OR 记忆障碍 OR 认知恢复延迟 OR 术后认知障碍 OR 术后认知功能障碍 OR 术后神经认知障碍 OR 术后认知并发症 OR 术后谵妄)  手动选择= (探索性研究/预试验/0 OR 随机平行对照/Parallel OR 干预性研究/Interventional study) | 55 |
| **ClinicalTrials.gov** | #1 | Condition/disease= (“neurocognitive disorders” OR “cognitive dysfunction” OR “cognition disorders” OR “cognitive disorders” OR “cognitive impairment” OR “cognitive decline” OR “cognition dysfunction” OR “cognition impairment” OR “cognition decline” OR “cognit*” OR “delirium” OR “postoperative cognitive complications” OR “delayed neurocognitive recovery” OR “postoperative cognitive dysfunction” OR “postoperative delirium” OR “perioperative neurocognitive disorder” OR “perioperative cognitive disorder” OR “POCD” OR “POD” OR “PND”) | 10 |
|  |  | Intervention/treatment= (“acupuncture” OR “electroacupuncture” OR “EA”)  Other terms= (“elderly” OR “older” OR “geriatric”)  Manually enter range= From “60” Years Old To “80” Years Old |  |
| [**http://itmctr.ccebtcm.org.cn/**](http://itmctr.ccebtcm.org.cn/) | #1 | Disease= (“neurocognitive disorders” OR “cognitive dysfunction” OR “cognition disorders” OR “cognitive disorders” OR “cognitive impairment” OR “cognitive decline” OR “cognition dysfunction” OR “cognition impairment” OR “cognition decline” OR “cognit*” OR “delirium” OR “postoperative cognitive complications” OR “delayed neurocognitive recovery” OR “postoperative cognitive dysfunction” OR “postoperative delirium” OR “perioperative neurocognitive disorder” OR “perioperative cognitive disorder” OR “POCD” OR “POD” OR “PND”)  Intervention= (“acupuncture” OR “electroacupuncture” OR “EA” | 12 |

**Supplementary Table S1.** (Continued)

**Supplementary Table S2.** Characteristics of included RCTs.

| **Studies** | **Sample size (EG/CG)** | **Age (mean ± SD/** **median (IQR)/range, years)** | **Gender (male/female)** | **Intervention** | | | | **Outcomes** | **Outcome evaluation timepoint** | **Diagnostic criteria for PND** | **Stimulus parameter** |
| --- | --- | --- | --- | --- | --- | --- | --- | --- | --- | --- | --- |
|  |  |  |  | **EA** | **Control** | **Acupoints** | **EA intervention timepoint and times** |  |  |  |  |
| Zhao et al. (2018) | 60(30/30) | EG: 65.23±4.03  CG: 66.70±3.84 | EG: 12/18  CG: 14/16 | EA | sham EA | Sishencong (EX-HN1), Shenting (GV24), Benshen (GB13), Baihui (GV20), Hegu (LI4), Taichong (LR3) | 5 days before surgery, once a day, 30 minutes/session | MMSE scores, incidence of POCD, serum IL-1β, TNF-α, and S100β levels, incidence of AEs | before operation; on POD^*^ 1 and POD^*^ 3 | a postoperative reduction of ≥ 2 points in MMSE score compared to preoperative baseline | dense-disperse wave; 2/100Hz; 3mA |
| Zhao et al. (2019) | 60(30/30) | EG:  67.53±4.93  CG:  68.20±5.18 | EG:  12/18  CG:  14/16 | EA | sham EA | Sishencong (EX-HN1), Shenting (GV24), Benshen (GB13), Baihui (GV20), Hegu (LI4), Taichong (LR3) | 5 days before surgery, once a day, 30 minutes/session | MMSE scores, incidence of POCD, serum IL-1β and TNF-α levels, incidence of AEs | before operation; on POD^*^ 1 | a postoperative reduction of ≥ 2 points in MMSE score compared to preoperative baseline | dense-disperse wave; based on patient tolerance |
| Wang et al. (2018) | 96(48/48) | EG:  68.74±5.57  CG:  68.36±5.43 | EG:  28/20  CG:  30/18 | EA | blank | Zusanli (ST36), Neiguan (PC6), Hegu (LI4), Shangjuxu (ST37) | 20 min before anesthesia induction | MMSE scores, MoCA scores, incidence of AEs | before operation; on POD^*^ 1 | postoperative MoCA score < 26 points; postoperative MMSE score < 24 points | dense wave; based on patient tolerance |
| Zheng et al. (2023) | 60(30/30) | EG:  70.5±1.5  CG:  70.6±1.4 | EG:  20/10  CG:  18/12 | EA | blank | Hegu (LI4), Taichong (LR3) | 3 days before and after surgery, once a day, 30 minutes/session; 30 min before operation to the end of surgery | MMSE scores, incidence of POCD | before operation; on POD^*^ 1, POD^*^ 3, and POD^*^ 14 | a postoperative reduction of ≥ 2 points in MMSE score compared to preoperative baseline | dense-disperse wave; 2/10Hz; based on patient tolerance |
| **studies** | **Sample size (EG/CG)**  **Supplementary Table S2.** (Continued) | **Age (mean ± SD/** **median (IQR)/range, years)** | **Gender (male/female)** | **Intervention** | | | | **Outcomes** | **Outcome evaluation timepoint** | **Diagnostic criteria for PND** | **Stimulus parameter** |
|  |  |  |  | **EA** | **Control** | **Acupoints** | **EA intervention timepoint and times** |  |  |  |  |
| Zhao et al. (2021) | 60(30/30) | EG:  71.63±4.72  CG:  71.53±4.59 | EG:  17/13  CG:  16/14 | EA | blank | Baihui (GV20), Shenmen (HT7), Neiguan (PC6), Hegu (LI4) | 5 days after surgery, once a day, 20 minutes/session | MMSE scores, incidence of POCD, serum IL-6 levels | before operation; on POD^*^ 1, POD^*^ 3, and POD^*^ 5 | NR | dense-disperse wave; 3/20Hz |
| Zhang et al. (2014) | 79(34/45) | EG:  67.5±4.7  CG:  70.1±5.2 | EG:  15/19  CG:  20/25 | EA | blank | Baihui (GV20), Shenting (GV24) | 30 min before surgery after anesthesia | incidence of POCD, serum IL-1β, IL-6, TNF-α, and S100β levels | before operation; at the end of surgery; on POD^*^ 1 and POD^*^ 7 | a postoperative reduction of ≥ 2 points in MMSE score compared to preoperative baseline | 2~15Hz; based on patient tolerance |
| Zhang et al. (2015) | 70(35/35) | EG:75±4  CG:74±4 | EG:  19/16  CG:  18/17 | EA | sham EA | Baihui (GV20), Neiguan (PC6) | after resuscitation from anesthesia and 7 days after surgery, once a day, 20 minutes/session | MMSE scores, incidence of POCD | before operation; on POD^*^ 1, POD^*^ 3, and POD^*^ 7 | a postoperative reduction of ≥ 2 points in MMSE score compared to preoperative baseline | dense-disperse wave; 4/20Hz; subtle vibration of the needle handle and/or mild muscle fasciculations at the acupoints |
| Yuan et al. (2019) | 100(50/50) | EG:61~79  CG:60~81 | EG:  26/24  CG:  27/23 | EA | sham EA | Baihui (GV20), Neiguan (PC6) | 20 min after removal of the endotracheal tube and 7 days after surgery, once a day, 20 minutes/session | MMSE scores, incidence of POCD | before operation; on POD^*^ 1, POD^*^ 3, and POD^*^ 7 | a postoperative reduction of ≥ 2 points in MMSE score compared to preoperative baseline | dense-disperse wave; 4/20Hz; mild muscle fasciculations at the acupoints |
| Yu et al. (2016) | 118(59/59) | EG:  75.6±4.1  CG:  75.8±4.0 | EG:  29/20  CG:  31/18 | EA | blank | Baihui (GV20), Neiguan (PC6), Zusanli (ST36), Sanyinjiao (SP6) | during the surgery, duration was not reported | MMSE scores, incidence of POCD | before anesthesia; at 6, 12, 24, and 72 hours postoperatively | a postoperative MMSE score of ≤ 23 points | NR |
| Yang et al. (2018) | 90(45/45) | EG:  75.71±8.4  CG:  74.7±8.9 | NR | EA | blank | Baihui (GV20), Neiguan (PC6), Zusanli (ST36) | 3 days before surgery, once a day, 30 minutes/session | incidence of POCD, serum NSE and S100β levels | before operation, on POD^*^ 1and POD^*^ 3 | a postoperative reduction of ≥ 2 points in MMSE score compared to baseline | dense-disperse wave; 2/100Hz; based on patient tolerance |
| **studies** | **Sample size (EG/CG)**  **Supplementary Table S2.** (Continued) | **Age (mean ± SD/** **median (IQR)/range, years)** | **Gender (male/female)** | **Intervention** | | | | **Outcomes** | **Outcome evaluation timepoint** | **Diagnostic criteria for PND** | **Stimulus parameter** |
|  |  |  |  | **EA** | **Control** | **Acupoints** | **EA intervention timepoint and times** |  |  |  |  |
| Yang et al. (2017) | 128(64/64) | EG:81±9  CG:82±8 | EG:  14/50  CG:  12/52 | EA | standard care | Shenting (GV24), Baihui (GV20), Neiguan (PC6) | 3 days before and after surgery, a total of 7 days, once a day, 20 minutes/session | incidence of POD, serum S100β levels | before operation; at the end of surgery; on POD^*^ 1, POD^*^ 2, and POD^*^ 3 | a postoperative Nu-DESC score of ≥ 2 points | dense-disperse wave; 2/20Hz; subtle vibration of the needle handle |
| Wu et al. (2020) | 167(86/81) | EG: 71(67~78)  CG: 73(67~77) | EG:  49/37  CG:  34/47 | EA | sham EA | Shenmen (HT7), Neiguan (PC6), Baihui (GV20), Yintang (EX-HN3) | 30 min before anesthesia induction to the end of surgery | incidence of POD, serum S100β levels | before anesthesia; at the end of surgery; on POD^*^ 1, POD^*^ 2, POD^*^ 3, POD^*^ 4, and POD^*^ 5 | CAM | dense-disperse wave; 2/100Hz; 1~30mA, based on patient tolerance |
| Wang et al. (2019) | 84(42/42) | EG:64~78  CG:63~79 | EG:  25/17  CG:  24/18 | EA | blank | Zusanli (ST36), Baihui (GV20), Neiguan (PC6) | 30 min before anesthesia induction to the end of surgery | MMSE scores, incidence of POCD, serum IL-6 and TNF-α levels | on preoperative day1; on POD^*^ 1 | a postoperative reduction of > 2 points in MMSE score compared to preoperative baseline | dense-disperse wave; 5~7.5mA, based on patient tolerance |
| Wan et al. (2023) | 60(30/30) | EG:74±9  CG:72±8 | EG:  18/12  CG:  21/9 | EA | blank | Zusanli (ST36), Neiguan (PC6) | 30 min before anesthesia induction | MMSE scores, incidence of POCD | before EA intervention; on POD^*^ 1, POD^*^ 3, and POD^*^ 7 | a postoperative reduction of ≥ 1 SD in MMSE score compared to preoperative baseline | dense-disperse wave; 2/100Hz; 1mA |
| Tang et al. (2022) | 60(30/30) | EG:  69.19±3.61  CG:  69.12±3.57 | EG:  15/15  CG:  17/13 | EA | blank | Yongquan (KI1), Shenmen (HT7), Baihui (GV20) | 30 min before anesthesia induction; 6 days after surgery, once a day, 30 minutes/session | MMSE scores, incidence of POCD, serum IL-6, TNF-α and S100β levels | at admission; on POD^*^ 1, POD^*^ 3, POD^*^ 5, and POD^*^ 7 | a postoperative reduction of ≥ 2 points in MMSE score compared to preoperative baseline | for the first 15 min: continuous wave; 2~100Hz; based on patient tolerance /within the next 15 min: dense-disperse wave; 2/100Hz; based on patient tolerance |
| **studies** | **Sample size (EG/CG)**  **Supplementary Table S2.** (Continued) | **Age (mean ± SD/** **median (IQR)/range, years)** | **Gender (male/female)** | **Intervention** | | | | **Outcomes** | **Outcome evaluation timepoint** | **Diagnostic criteria for PND** | **Stimulus parameter** |
|  |  |  |  | **EA** | **Control** | **Acupoints** | **EA intervention timepoint and times** |  |  |  |  |
| Liu Z et al. (2018) | 99(50/49) | EG:  68.87±12.31  CG:  67.94±10.20 | EG:  24/26  CG:  25/24 | EA | blank | Baihui (GV20), Neiguan (PC6), Zusanli (ST36), Sanyinjiao (SP6) | 20 min before anesthesia induction to the end of surgery | MMSE scores, incidence of POCD, serum NSE and S100β levels | on preoperative day1; at the end of surgery; at 1hour postoperatively; on POD^*^ 1 | a postoperative MMSE score of < 23 points | dense-disperse wave; 4/20Hz; 5mA, based on patient tolerance |
| Liu et al. (2017) | 98(49/49) | EG:  73.61±11.18  CG:  74.26±10.38 | EG:  25/24  CG:  23/26 | EA | blank | Baihui (GV20), Neiguan (PC6), Zusanli (ST36) | 30 min before anesthesia induction to the end of surgery | MMSE scores, incidence of POCD, serum TNF-α, IL-1β and IL-6 levels | on preoperative day1; at the end of surgery; on POD^*^ 1, POD^*^ 2, and POD^*^ 3 | a postoperative MMSE score of ≤ 27 points | dense-disperse wave; 2/100Hz; based on patient tolerance |
| Liu et al. (2023) | 120(60/60) | EG:  72.47±5.82  CG:  72.15±5.67 | EG:  27/33  CG:  29/31 | EA | blank | Daimai (GB26), Ashixue, Zulinqi (GB41), Fengshi (GB31) | 30 min before anesthesia induction to the end of surgery | MMSE scores, serum NSE and S100β levels, incidence of AEs | before surgery; at the end of surgery; at 1 hour postoperatively; on POD^*^ 1 | a postoperative MMSE score of < 23 points | based on patient tolerance |
| Liu P et al. (2018) | 120(60/60) | EG:65±8  CG:66±5 | EG:  25/35  CG:  26/34 | EA | blank | Hegu (LI4), Taichong (LR3) | 3 days before and after surgery, once a day, 30 minutes/session; 30 min before operation to the end of surgery | MMSE scores, incidence of POCD, serum IL-1β and TNF-α levels | before surgery; at 1, 6, 12, and 24 hours postoperatively; on POD^*^ 1, POD^*^ 2, POD^*^ 3, and POD^*^ 4 | a postoperative reduction of ≥ 2 points in MMSE score compared to preoperative baseline | dense-disperse wave; 2/10Hz; based on patient tolerance |
| Lin et al. (2014) | 83(42/41) | EG: 68.4±3.2  CG: 67.5±2.9 | EG: 28/14  CG: 26/15 | EA | blank | Baihui (GV20), Neiguan (PC6), Zusanli (ST36) | 30 min before anesthesia induction to the end of surgery | MMSE scores, incidence of POCD | on preoperative day1; at the end of surgery; on POD^*^ 1, POD^*^ 2, and POD^*^ 3 | a postoperative reduction of ≥ 1 SD in MMSE score compared to preoperative baseline | dense-disperse wave; 2/100Hz; based on patient tolerance |
| **studies** | **Sample size (EG/CG)** | **Age (mean ± SD/** **median (IQR)/range, years)** | **Gender (male/female)** | **Intervention** | | | | **Outcomes** | **Outcome evaluation timepoint** | **Diagnostic criteria for PND** | **Stimulus parameter** |
|  |  |  |  | **EA** | **Control** | **Acupoints** | **EA intervention timepoint and times** |  |  |  |  |
| Lin et al. (2013) | 75(38/37) | EG: 69±4  CG: 68±3 | EG: 25/13  CG: 23/14 | EA | blank | Baihui (GV20), Neiguan (PC6), Zusanli (ST36), Sanyinjiao (SP6) | 20 min before anesthesia induction to the end of surgery | MMSE scores, incidence of POCD, serum S100β levels | on preoperative day1; at the end of surgery; on POD^*^ 3 | a postoperative reduction of ≥ 1 SD in MMSE score compared to preoperative baseline | dense-disperse wave; 4/20Hz; based on patient tolerance |
| Han et al. (2018) | 90(45/45) | EG:  68.52±2.44  CG:  67.93±2.60 | EG:  26/19  CG:  24/21 | EA | blank | Baihui (GV20), Sanyinjiao (SP6), Zusanli (ST36), Neiguan (PC6) | 20 min before anesthesia induction to the end of surgery | MMSE scores, incidence of POCD, serum S100β levels | before anesthesia; at 6, 12, 24, 36, and 72 hours postoperatively | a postoperative MMSE score of ≤ 23 points | dense-disperse wave; 4/20Hz; based on patient tolerance |
| Gu et al. (2022) | 64(32/32) | EG:  73.37±6.39  CG:  72.25±6.21 | EG:  16/16  CG:  15/17 | EA | blank | Neiguan (PC6), Baihui (GV20), Hegu (LI4), Sishencong (EX-HN1), Zusanli (ST36) | 1 day before surgery, 30 minutes/session; 30 min before anesthesia induction to the end of surgery | MMSE scores, incidence of AEs | before surgery; on POD^*^ 1 | a postoperative MMSE score of ≤ 24 points | dense-disperse wave; 0.02Hz; 2mA |
| Gao et al. (2012) | 120(60/60) | EG:  72.2±4.3  CG:  71.1±5.3 | EG:  28/32  CG:  24/36 | EA | blank | Baihui (GV20), Hegu (LI4), Neiguan (PC6), Zusanli (ST36) | 30 min before anesthesia induction to the end of surgery | MMSE scores, incidence of POCD, incidence of AEs | 1 day before anesthesia; on POD^*^ 2, POD^*^ 4, and POD^*^ 6 | a postoperative MMSE score of ≤ 27 points | dense-disperse wave; 2/100Hz; based on patient tolerance |
| Dong et al. (2016) | 60(30/30) | EG:71±5  CG:69±4 | NR | EA | blank | Baihui (GV20), Neiguan (PC6) | 30 min before anesthesia induction to the end of surgery | MMSE scores, incidence of POCD, incidence of AEs | 1 day before anesthesia; on POD^*^ 1, POD^*^ 3, and POD^*^ 7 | a postoperative reduction of ≥ 2 points in MMSE score compared to preoperative baseline | dense-disperse wave; 2/100Hz; based on patient tolerance |
| Zhang et al. (2011) | 88(41/47) | EG:  74.2±5.9  CG:  75.0±6.5 | EG:  19/22  CG:  20/27 | EA | blank | Baihui (GV20), Shenting (GV24) | 30 min before anesthesia induction | MMSE scores, incidence of POCD, serum S100β levels, incidence of AEs | on preoperative day1; at the end of surgery; on POD^*^ 1 and POD^*^ 7 | a postoperative reduction of ≥ 2 points in MMSE score compared to baseline | 2~15Hz; based on patient tolerance |

**Supplementary Table S2.** (Continued)

EG, electroacupuncture group; CG, control group; SD, standard deviation; IQR, interquartile range; EA, electroacupuncture; PND, perioperative neurocognitive disorder; POCD, postoperative cognitive dysfunction; POD, postoperative delirium; MMSE, Mini-Mental State Examination; AEs, adverse events; MoCA, Montreal Cognitive Assessment; IL-1β, interleukin-1β; IL-6, interleukin-6; TNF-α, tumor necrosis factor-α; NSE, neuron-specific enolase; S100β, S100 calcium-binding protein β; POD^*^, postoperative day; Nu-DESC, Nursing Delirium Screening Scale; CAM, Confusion Assessment Method; NR, not reported.

**Supplementary Table S3.** Meta-analysis for the serum IL-6, IL-1β, TNF-α, and S100β levels at different periods.

| **Outcomes** | **No. of Studies** | **No. of Participants** | | | **Effect Size (95% CI)** | ***p*** | **I^2^ (%)** | |  |
| --- | --- | --- | --- | --- | --- | --- | --- | --- | --- |
|  |  | **EG** | **CG** | |  |  |  |  |  |
| **IL-6** | | | | | | | |  |  |
| at the end of surgery | 2 | 83 | | 94 | SMD -0.72 (-1.89~0.44) | = 0.22 | 93 | |  |
| on postoperative day 1 | | 5 | 185 | | 196 | SMD -1.09 (-1.73~-0.44) | = 0.0010 | 88 | |
| **IL-1β** | | | | | | | |  |  |
| at the end of surgery | 2 | 83 | | 94 | SMD -1.04 (-2.69~0.61) | = 0.22 | 96 | |  |
| on postoperative day 1 | 4 | 173 | | 184 | SMD -2.85 (-5.32~-0.39) | = 0.02 | 99 | |  |
| **TNF-α** | | | | | | | |  |  |
| on postoperative day 1 | 6 | 245 | | 256 | SMD -2.64 (-4.16~-1.12) | = 0.0007 | 98 | |  |
| on postoperative day 3 | 2 | 60 | | 60 | SMD -3.70 (-7.96~0.55) | = 0.09 | 98 | |  |
| **S100β** | | | | | | | |  |  |
| at the end of surgery | 4 | 208 | | 212 | SMD -0.58 (-1.29~0.13) | = 0.11 | 92 | |  |
| on postoperative day 1 | 6 | 275 | | 280 | SMD -1.56 (-2.77~-0.35) | = 0.01 | 97 | |  |

IL-6, interleukin-6; IL-1β, interleukin-1β; TNF-α, tumor necrosis factor-α; S100β, S100 calcium-binding protein β; EG, electroacupuncture group; CG, control group; CI, confidence intervals; SMD, standardized mean difference.

**Supplementary Table S4.** Subgroup analyses.

| **Outcomes** | **No. of Studies** | **No. of Participants** | **Effect Size**  **(95% CI)** | ***p*** | **I^2^ (%)** | **GRADE** |
| --- | --- | --- | --- | --- | --- | --- |
| **PND incidence on postoperative day 1** | | | | | | |
| preoperative EA​​ | 6 | 437 | RR 0.55 (0.39~0.77) | = 0.0004 | 0 | moderate |
| intraoperative EA​​ | 5 | 439 | RR 0.42 (0.28~0.63) | < 0.0001 | 0 | low |
| postoperative EA​​ | 2 | 170 | RR 0.61 (0.43~0.87) | = 0.006 | 0 | low |
| preoperative-postoperative combined EA | 3 | 248 | RR 0.49 (0.32~0.75) | = 0.001 | 0 | low |
| **PND incidence on postoperative day 1** | | | | | | |
| sham EA control | 4 | 290 | RR 0.64 (0.47~0.86) | = 0.004 | 0 | moderate |
| no-intervention control | 11 | 876 | RR 0.46 (0.36~0.59) | < 0.00001 | 0 | moderate |
| standard care control | 1 | 128 | RR 0.54 (0.23~1.26) | = 0.15 | NA | low |
| **PND incidence on postoperative day 1** | | | | | | |
| orthopedic surgery | 8 | 619 | RR 0.50 (0.38~0.66) | < 0.00001 | 0 | moderate |
| urological surgery | 2 | 150 | RR 0.50 (0.25~1.00) | = 0.05 | 0 | low |
| gastrointestinal surgery | 5 | 426 | RR 0.57 (0.43~0.75) | < 0.0001 | 0 | moderate |
| multiple surgical types | 1 | 99 | RR 0.22 (0.05~0.96) | = 0.04 | NA | low |
| **PND incidence on postoperative day 3** | | | | | | |
| preoperative EA​​ | 3 | 210 | RR 0.46 (0.26~0.83) | = 0.01 | 0 | low |
| intraoperative EA​​ | 6 | 504 | RR 0.52 (0.37~0.75) | = 0.0003 | 0 | moderate |
| postoperative EA​​ | 2 | 170 | RR 0.33 (0.16~0.70) | = 0.004 | 0 | low |
| preoperative-postoperative combined EA | 3 | 248 | RR 0.22 (0.10~0.48) | = 0.0002 | 24 | low |
| **PND incidence on postoperative day 3** | | | | | | |
| sham EA control | 3 | 230 | RR 0.45 (0.27~0.74) | = 0.002 | 0 | low |
| no-intervention control | 10 | 774 | RR 0.44 (0.32~0.60) | < 0.00001 | 0 | moderate |
| standard care control | 1 | 128 | RR 0.06 (0.00~1.00) | = 0.05 | NA | low |
| **PND incidence on postoperative day 3** | | | | | | |
| orthopedic surgery | 4 | 308 | RR 0.36 (0.21~0.59) | < 0.0001 | 47 | low |
| urological surgery | 2 | 150 | RR 0.25 (0.07~0.84) | = 0.03 | 0 | low |
| gastrointestinal surgery | 8 | 674 | RR 0.47 (0.34~0.65) | < 0.00001 | 0 | moderate |
| **Outcomes** | **No. of Studies** | **No. of Participants** | **Effect Size**  **(95% CI)** | ***p*** | **I^2^ (%)** | **GRADE** |
| **MMSE scores on postoperative day 1** | | | | | | |
| preoperative EA​​ | 5 | 364 | MD 1.59 (0.48~2.69) | = 0.005 | 83 | very low |
| intraoperative EA​​ | 7 | 649 | MD 1.85 (1.38~2.33) | < 0.00001 | 93 | low |
| postoperative EA​​ | 3 | 230 | MD 4.20 (1.17~7.22) | = 0.007 | 97 | very low |
| preoperative-intraoperative combined EA | 1 | 64 | MD 2.29 (1.53, 3.05) | < 0.00001 | NA | low |
| preoperative-postoperative combined EA | 2 | 120 | MD 3.51 (2.64~4.37) | < 0.00001 | 0 | low |
| **MMSE scores on postoperative day 3** | | | | | | |
| preoperative EA​​ | 2 | 120 | MD 1.84 (1.05, 2.64) | < 0.00001 | 0 | low |
| intraoperative EA​​ | 6 | 504 | MD 1.69 (0.45, 2.92) | = 0.007 | 95 | low |
| postoperative EA​​ | 3 | 230 | MD 3.50 (2.94, 4.06) | < 0.00001 | 0 | low |
| preoperative-postoperative combined EA | 2 | 120 | MD 3.07 (2.40, 3.74) | < 0.00001 | 0 | low |
| **serum IL-6 levels on postoperative day 1** | | | | | | |
| a single EA session | 3 | 261 | SMD -0.73 (-1.54~0.07) | = 0.07 | 90 | very low |
| multiple EA sessions (≥2) | 2 | 120 | SMD -1.65 (-2.07~-1.23) | < 0.00001 | 0 | low |
| **serum IL-1β levels on postoperative day 1** | | | | | | |
| a single EA session | 2 | 177 | SMD -0.29 (-1.35~0.77) | = 0.59 | 92 | very low |
| multiple EA sessions (≥2) | 2 | 180 | SMD -5.37 (-6.09~-4.65) | < 0.00001 | 17 | low |
| **serum TNF-α levels on postoperative day 1** | | | | | | |
| preoperative EA​​ | 2 | 139 | SMD -3.93 (-12.07. 4.22) | = 0.34 | 99 | very low |
| intraoperative EA​​ | 2 | 182 | SMD -1.07 (-1.43~-0.72) | < 0.00001 | 22 | low |
| preoperative-postoperative combined EA | 1 | 60 | SMD -1.52 (-2.10, -0.94) | < 0.00001 | NA | low |
| preoperative-intraoperative-postoperative combined EA | 1 | 120 | SMD -5.08 (-5.83, -4.34) | < 0.00001 | NA | low |
| **serum S100β levels on postoperative day 1** | | | | | | |
| orthopedic surgery | 3 | 199 | SMD -0.21 (-0.56~0.13) | = 0.22 | 33 | low |
| urological surgery | 1 | 90 | SMD -1.33 (-1.79, -0.87) | < 0.00001 | NA | low |
| multiple surgical types | 2 | 266 | SMD -3.71 (-5.91~-1.51) | = 0.0009 | 96 | very low |

CI, confidence intervals; GRADE, Grading of Recommendations, Assessment, Development and Evaluation; PND, perioperative neurocognitive disorder; EA, electroacupuncture; RR, relative risk; NA, not applicable; MMSE, Mini-Mental State Examination; MD, mean difference; IL-6, interleukin-6; SMD, standardized mean difference; IL-1β, interleukin-1β; TNF-α, tumor necrosis factor-α; S100β, S100 calcium-binding protein β.

**Supplementary Table S4.** (Continued)

**Supplementary Table S4.** (Continued)

Supplementary Table S5. Sensitivity analysis for PND incidence and MMSE scores.

| **Outcomes** | **References** | **Effect Size** | **95% CI** | **Z** | ***p*** | **I^2^ (%)** |
| --- | --- | --- | --- | --- | --- | --- |
| incidence of PND on postoperative day 1 | Yang et al. (2017) | 0.51 | 0.42, 0.62 | 6.75 | < 0.00001 | 0 |
|  | Wan et al. (2023) | 0.52 | 0.43, 0.63 | 6.58 | < 0.00001 | 0 |
|  | Liu et al. (2017) | 0.52 | 0.43, 0.64 | 6.21 | < 0.00001 | 0 |
|  | Liu Z et al. (2018) | 0.54 | 0.44, 0.66 | 5.87 | < 0.00001 | 0 |
|  | Zhang et al. (2011) | 0.55 | 0.44, 0.68 | 5.52 | < 0.00001 | 0 |
|  | Zhang et al. (2015) | 0.54 | 0.42, 0.68 | 5.12 | < 0.00001 | 0 |
|  | Yang et al. (2018) | 0.54 | 0.42, 0.69 | 4.96 | < 0.00001 | 0 |
|  | Tang et al. (2022) | 0.53 | 0.40, 0.71 | 4.37 | < 0.0001 | 0 |
|  | Wang et al. (2019) | 0.57 | 0.43, 0.76 | 3.82 | = 0.0001 | 0 |
|  | Dong et al. (2016) | 0.56 | 0.40, 0.79 | 3.30 | = 0.0010 | 0 |
|  | Zheng et al. (2023) | 0.60 | 0.43, 0.85 | 2.87 | = 0.004 | 0 |
|  | Yu et al. (2016) | 0.61 | 0.43, 0.86 | 2.81 | = 0.005 | 0 |
| incidence of PND on postoperative day 3 | Yang et al. (2017) | 0.44 | 0.34, 0.58 | 6.01 | < 0.00001 | 0 |
|  | Wan et al. (2023) | 0.45 | 0.34, 0.59 | 5.78 | < 0.00001 | 0 |
|  | Liu et al. (2017) | 0.46 | 0.35, 0.61 | 5.47 | < 0.00001 | 0 |
|  | Yang et al. (2018) | 0.47 | 0.36, 0.62 | 5.26 | < 0.00001 | 0 |
|  | Lin et al. (2013) | 0.44 | 0.33, 0.60 | 5.20 | < 0.00001 | 0 |
|  | Lin et al. (2014) | 0.41 | 0.29, 0.58 | 5.10 | < 0.00001 | 0 |
|  | Tang et al. (2022) | 0.41 | 0.29, 0.60 | 4.66 | < 0.00001 | 0 |
|  | Dong et al. (2016) | 0.44 | 0.29, 0.65 | 4.06 | < 0.0001 | 0 |
|  | Zheng et al. (2023) | 0.48 | 0.32, 0.73 | 3.49 | = 0.0005 | 0 |
|  | Han et al. (2018) | 0.46 | 0.28, 0.75 | 3.09 | = 0.002 | 0 |
|  | Yu et al. (2016) | 0.45 | 0.27, 0.74 | 3.16 | = 0.002 | 0 |
| MMSE scores on postoperative day 1 | Wang et al. (2018) | 2.36 | 1.84, 2.88 | 8.87 | < 0.00001 | 94 |
|  | Wan et al. (2023) | 2.40 | 1.86, 2.95 | 8.68 | < 0.00001 | 95 |
|  | Liu et al. (2023) | 2.45 | 1.82, 3.09 | 7.58 | < 0.00001 | 95 |
|  | Liu et al. (2017) | 2.47 | 1.80, 3.13 | 7.25 | < 0.00001 | 95 |
|  | Liu Z et al. (2018) | 2.53 | 1.66, 3.39 | 5.72 | < 0.00001 | 96 |
|  | Zhang et al. (2011) | 2.56 | 1.65, 3.47 | 5.53 | < 0.00001 | 96 |
|  | Tang et al. (2022) | 2.45 | 1.51, 3.39 | 5.12 | < 0.00001 | 96 |
|  | Wang et al. (2019) | 2.54 | 1.53, 3.54 | 4.93 | < 0.00001 | 97 |
|  | Dong et al. (2016) | 2.37 | 1.33, 3.41 | 4.46 | < 0.00001 | 97 |
|  | Zhao et al. (2021) | 2.54 | 1.42, 3.67 | 4.43 | < 0.00001 | 97 |
|  | Zheng et al. (2023) | 2.45 | 1.24, 3.65 | 3.98 | < 0.0001 | 97 |
|  | Han et al. (2018) | 2.60 | 0.53, 4.66 | 2.46 | = 0.01 | 98 |
|  | Gu et al. (2022) | 2.66 | 0.05, 5.26 | 2.00 | = 0.05 | 98 |
|  | Yu et al. (2016) | 3.19 | 0.20, 6.17 | 2.09 | = 0.04 | 97 |
| MMSE scores on postoperative day 3 | Wan et al. (2023) | 2.37 | 1.41, 3.32 | 4.85 | < 0.00001 | 94 |
| **Outcomes** | **References** | **Effect Size** | **95% CI** | **Z** | ***p*** | **I^2^ (%)** |
| MMSE scores on postoperative day 3 | Liu et al. (2017) | 2.37 | 1.31, 3.43 | 4.37 | < 0.0001 | 95 |
|  | Lin et al. (2013) | 2.42 | 1.26, 3.57 | 4.10 | < 0.0001 | 95 |
|  | Lin et al. (2014) | 2.38 | 1.13, 3.62 | 3.73 | = 0.0002 | 95 |
|  | Tang et al. (2022) | 2.26 | 0.94, 3.58 | 3.36 | = 0.0008 | 96 |
|  | Dong et al. (2016) | 2.11 | 0.71, 3.50 | 2.96 | = 0.003 | 96 |
|  | Zhao et al. (2021) | 1.91 | 0.43, 3.39 | 2.54 | = 0.01 | 96 |
|  | Zheng et al. (2023) | 1.73 | 0.11, 3.34 | 2.10 | = 0.04 | 96 |
|  | Han et al. (2018) | 2.15 | -0.16, 4.47 | 1.82 | = 0.07 | 97 |
|  | Yu et al. (2016) | 3.07 | 2.04, 4.10 | 5.86 | < 0.00001 | 65 |

PND, perioperative neurocognitive disorder; MMSE, Mini-Mental State Examination; CI, confidence intervals.

**Supplementary Table S5.** (Continued)

**Supplementary Table S5.** (Continued)

**
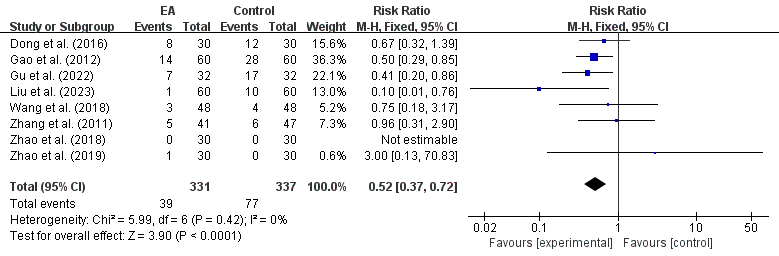
**

Supplementary Figure 1. Meta-analysis and forest plot for the incidence of adverse events. EA, electroacupuncture; CI, confidence intervals.
